# Decitabine Reprograms Temozolomide-Resistant Glioblastoma Through Epigenetic Reactivation and Mesenchymal Attenuation: A Multi-Omics Study

**DOI:** 10.3390/cancers18162616

**Published:** 2026-08-14

**Authors:** Itika Arora, Shamsa Hilal Saleh, Arshiya Akbar, Fareeha Arshad, Volodymyr Mavrych, Olena Bolgova, Faisal Abdulhameed Farrash, Ahmed Abu-Zaid, Andleeb Khan, Sheikh Muskan, Mohammed Imran Khan, Ahmed Yaqinuddin

**Affiliations:** 1 College of Medicine, Alfaisal University, Riyadh 11533, Saudi Arabia; 2 King Faisal Specialist Hospital and Research Centre (KFSHRC), Riyadh 12713, Saudi Arabia; 3 Department of Biosciences, Faculty of Science, Integral University, Lucknow 226026, India; 4 Stem Cell Biology Lab, Integral Center of Excellence for Interdisciplinary Research-4 (ICEIR-12), Integral University, Lucknow 226026, India; 5 King Faisal Specialist Hospital and Research Centre (KFSHRC), Jeddah 23433, Saudi Arabia

**Keywords:** temozolomide resistance, decitabine, epigenetic de-repression, INPP5D/SHIP1, PI3K–AKT signaling, multi-omics integration, single-cell transcriptomics, TCGA-GBM survival, pharmacogenomics, interferon response, mesenchymal transition

## Abstract

**Background/Objectives**: Glioblastoma (GBM) is the most lethal primary brain malignancy in adults, with a median overall survival of approximately 15 months. Temozolomide (TMZ) resistance develops in virtually all patients, and no second-line regimen has improved outcomes over the past two decades. The DNA methyltransferase inhibitor decitabine (DAC) has attracted interest as a chemosensitizer, but whether it directly reverses the TMZ-resistance transcriptome or operates through distinct, complementary mechanisms has not been tested at multi-omics resolution. **Methods**: We performed an integrative six-layer multi-omics analysis across five public GEO datasets (bulk RNA-seq, EPIC 850K methylation, and 21,676 single cells) re-purposed from studies conducted for unrelated aims, formally tested DAC-mediated reversal of the TMZ-resistance transcriptome across 11,707 genes, mapped pharmacogenomic targets with DGIdb v5, and built an exploratory, hypothesis-generating 11-gene prognostic model internally validated in TCGA-GBM (n = 166) and externally tested in the independent CPTAC-GBM cohort (n = 96). **Results**: DAC reprogrammed transcription across 1114–1882 differentially expressed genes per cohort and reactivated 146 direct epigenetic targets, identifying INPP5D/SHIP1 as the top-ranked direct epigenetic-reactivation target. Genome-wide reversal analysis across 11,707 co-detected genes showed a negligible effect (Spearman ρ = 0.073), but single-cell analysis revealed significant per-cell attenuation of MES-like and stem-like programs (Δ = −0.071 and −0.135, respectively; both *p* < 0.001). The 11-gene risk model achieved a Harrell’s C-index of 0.706 (apparent); after correcting for the two-stage gene selection with a full-pipeline bootstrap, the optimism-corrected C-index was 0.63, and external validation in an independent cohort (CPTAC-GBM, n = 96) showed only near-chance discrimination (C-index 0.55), indicating that the signature does not generalize and is exploratory. Pharmacogenomic mapping yielded 734 unique therapeutic agents (230 FDA-approved) across 69 druggable targets after excluding AR. Most of these agents are not GBM-directed, so this catalog-level mapping is hypothesis-generating rather than a set of therapeutic recommendations. **Conclusions**: DAC does not broadly reverse the TMZ-resistant transcriptome but acts through three complementary mechanisms: epigenetic reactivation of INPP5D/SHIP1, cancer-testis-antigen and type I interferon induction, and per-cell attenuation of mesenchymal–stem-like transcriptional intensity, supporting hypotheses for rationally designed DAC-based combination therapy in TMZ-resistant GBM.

## Simple Summary

Glioblastoma is the deadliest brain cancer, and tumors almost always become resistant to the standard chemotherapy drug temozolomide. Decitabine, a drug already approved for blood cancers, has been proposed to make resistant tumors respond again, but it was unclear whether decitabine works by directly undoing the changes that cause resistance or through separate mechanisms. We combined five public datasets covering gene activity, DNA modification, and single-cell profiles of glioblastoma cells to test this directly. We found that decitabine does not broadly reverse the resistance pattern; instead, it reawakens a silenced gene called INPP5D, reduces the aggressive, stem-like character of resistant cells, and switches on an immune-alerting, antiviral-like response. From these findings we built an exploratory eleven-gene tool that, in a single dataset, separates patients by likely survival outcome. These results point to specific drug combinations worth testing together with decitabine in resistant glioblastoma.

## 1. Introduction

GBM is the most aggressive primary malignant brain tumor in adults, with a median overall survival of approximately 15 months despite multimodal therapy [1,2]. Acquired TMZ resistance develops in virtually all patients and extends beyond MGMT promoter methylation [3], reflecting a multidimensional cellular state shaped by myeloid differentiation programs, NF-κB-dependent mesenchymal transition, metabolic reprogramming, and paradoxical type I interferon responses that promote tumor survival rather than cytotoxic immunity [4,5,6]. This complexity motivates epigenetic approaches that can simultaneously reshape multiple resistance-associated programs. GBM is also characterized by widespread aberrant epigenetic regulation, including promoter hypermethylation that silences tumor-suppressive and immune-modulatory loci [7,8]. Decitabine (DAC, 5-aza-2′-deoxycytidine) is an FDA-approved DNA methyltransferase (DNMT) inhibitor with established clinical activity in hematological malignancies. In addition to its demethylating activity, DAC can trigger a ‘viral mimicry’ response in which derepression of endogenous retroviral elements (ERVs) and double-stranded RNA activates cytosolic nucleic acid-sensing pathways, stimulating innate immune signaling [9,10]. Prior studies have also demonstrated that DAC-mediated demethylation of the MLH1 promoter can restore mismatch repair (MMR) competence and potentiate TMZ cytotoxicity through synthetic lethality [11] and that DAC-mediated re-expression of cancer testis antigens (CTAs) and neoantigens sensitizes GBM cells to T-cell-mediated killing [12]. Despite these mechanistic rationales, the transcriptomic, epigenomic, and single-cell dimensions through which DAC exerts its effects in established TMZ resistance have not been systematically characterized at multi-omics resolution.

Beyond that, the extensive cellular heterogeneity of GBM, now recognized as a central driver of tumor evolution and therapeutic response [13], has not been systematically examined in relation to TMZ resistance and DAC treatment at single-cell resolution. To address these gaps, we conducted an integrative multi-omics analysis across five independent GEO datasets, encompassing bulk RNA sequencing, EPIC 850K DNA methylation profiling, and single-cell transcriptomics (21,676 cells). To test whether DAC reverses the TMZ-resistant transcriptome, we applied a six-layer evidence-integration framework. We performed a formal quantitative test of the reversal hypothesis across 11,707 genes and conducted internal validation using TCGA-GBM survival data (n = 166 patients) [14] with external validation in an independent CPTAC-GBM cohort, pharmacogenomic mapping via DGIdb v5 [15], and machine learning approaches. Together, these analyses provide an integrated view of how DAC acts in TMZ-resistant GBM and inform the rationale for combination strategies. In brief, this study addresses a single, clinically motivated question: in established TMZ resistance, does decitabine reverse the resistance transcriptome, or does it act through separate mechanisms that could instead inform rational combination therapy?

The five GEO datasets analyzed here were originally generated for different purposes than those pursued in the present study. GSE261187, GSE261188, and GSE261190 were generated by Lai et al., who characterized DAC-induced reactivation of cancer testis antigens and endogenous retroviruses, and consequent interferon induction, in glioma at single-cell resolution, establishing a rationale for DAC as an immunotherapy-sensitizing agent [16]. GSE151680 was generated by Liu et al., who used TMZ-resistant U87MG/U251MG RNA-sequencing data, together with CGGA, TCGA, and REMBRANDT cohorts, to construct and externally validate a ferroptosis-related prognostic gene signature in glioma [17]. Neither study addressed the question motivating the present work: whether DAC directly reverses the transcriptional program underlying established TMZ resistance or instead acts through mechanistically distinct programs. Lai et al. did not analyze TMZ-resistance data or formally test transcriptional reversal against a resistance signature; Liu et al. did not analyze DAC-treatment, methylation, or single-cell data, and their ferroptosis-focused signature is thematically and analytically independent of the six-layer, DAC-response-derived evidence framework used here. The present study instead re-purposes and integrates these five independent datasets, together with EPIC 850K methylation, single-cell transcriptomics, TCGA-GBM survival data, and DGIdb pharmacogenomic mapping not analyzed in either source publication, to formally test the reversal hypothesis across 11,707 genes, define direct epigenetic-reactivation targets (including INPP5D/SHIP1), and derive and internally validate an independent, DAC-response-derived prognostic signature. This six-layer integrative, hypothesis-testing framework, rather than the individual datasets themselves, constitutes the principal advance of the present work over Lai et al. (2024) [16] and Liu et al. (2020) [17].

## 2. Materials and Methods

### 2.1. Public Datasets and Data Accessibility

All data were sourced from publicly available repositories. The datasets analyzed here are listed below (Table 1):

Raw data were downloaded from Gene Expression Omnibus (GEO [18]; https://www.ncbi.nlm.nih.gov/geo/, accessed on 10 July 2026) using the accession IDs listed above. GSE151680 was originally generated by Liu et al. [17] to construct and externally validate a ferroptosis-related prognostic gene signature in glioma, and GSE261187, GSE261188, and GSE261190 were originally generated by Lai et al. [16] to characterize DAC-induced cancer testis antigen and endogenous retrovirus reactivation at single-cell resolution; neither prior study analyzed TMZ resistance or tested transcriptional reversal by DAC (see Section 1). TCGA-GBM data were accessed via UCSC Xena (https://xenabrowser.net/, accessed on 10 July 2026). All analyses comply with data usage agreements for publicly available, de-identified data.

### 2.2. Bulk RNA-Sequencing Analysis

Raw RNA-sequencing reads were aligned and quantified using standard pipelines (STAR aligner with HTSeq for GSE151680 and GSE261187, and featureCounts for GSE80137) [19,20]. Count matrices were imported into R (v4.3.0) and normalized using the trimmed mean of M-values (TMM) method implemented in edgeR [21]. Differential expression analysis was performed with limma-trend [22] on TMM-normalized log-CPM values. Variance estimates were stabilized using empirical Bayes moderation. Genes with FDR < 0.05 and |log_2_FC| > 1 were considered significant. GSEA was performed using the fgsea package (v1.22.0) [23] with MSigDB Hallmark gene sets (all 50 pathways tested; 10,000 gene-level permutations; signal-to-noise ratio ranking for TMZ (GSE151680: n = 6 per group across both U87MG and U251MG), log_2_FC ranking for DAC (GSE261187: n = 3 per group; GSE80137: n = 4 per group) [24]. Gene-level permutation was used per Subramanian et al. recommendations for n < 7 per group; Hallmark pathway annotations are in Supplementary Table S2. Transcription factor activity was inferred using DoRothEA (dorothea R package, v1.8.0) [25] with confidence level A and B regulons, using the VIPER algorithm for activity estimation on human regulons [26]. TFs with BH-adjusted *p* < 0.05 were considered significantly active.

### 2.3. Pathway Enrichment and Network Analysis

Functional enrichment analysis used clusterProfiler (v4.2.0) for Gene Ontology, KEGG, and Reactome pathways (BH-corrected, q < 0.05). GSVA calculated pathway activity scores per sample. Gene regulatory networks were inferred using GENIE3 [27] (random forest regression) and visualized using igraph. Given the small sample sizes available for network inference (n = 8–12), the GENIE3 network should be considered exploratory; the standard recommendation for reliable network inference is ≥50 samples per condition, and the identified hub genes (e.g., JUN) may partly reflect known transcription factor biology rather than de novo regulatory relationships. SNR ranking was used for the small-sample TMZ cohort (GSE151680, n = 6 per group); log_2_FC ranking for the larger DAC datasets. Headline pathway results were robust to the choice of ranking metric.

### 2.4. DNA Methylation Analysis

EPIC 850K array data (GSE261190) were processed in minfi (v1.42.0) [28] with Noob background correction, dye-bias normalization, and BMIQ type I/II probe bias adjustment [29]. Probe filtering removed probes with detection *p* > 0.01 (n = 2415), probes with <3 beads in >5% of samples (n = 1834), sex chromosome probes (n = 19,681), SNP-overlapping probes (n = 35,214), and cross-reactive probes (n = 43,254; [30]), retaining 803,785 of 865,918 probes (net unique removal = 62,133; category overlap counts in Supplementary Table S3, since filter categories are non-exclusive). Differential methylation analysis used limma on M-values; significant DMPs were defined by adjusted *p* < 0.05 and |Δβ| > 0.1.

### 2.5. Methylation–Expression Integration

DMPs were mapped to promoter regions (±2 kb from the TSS) and to gene-body, UTR, and enhancer regions using EPIC array annotations. Probe-to-gene mapping yielded 14,441 unique genes with at least one EPIC probe co-detected in the matched RNA-seq expression data; these 14,441 genes constitute the methylation–expression integration background. For each gene, mean Δβ across promoter probes, log_2_FC from expression data, and a composite epigenetic score (epi_score = |Δβ| × |log_2_FC|) were calculated. Genes were categorized by directionality (Hypo + Up or Hyper + Down), yielding 146 direct epigenetic targets. To assess potential microglial contamination of the GSC cultures, an in silico marker-based deconvolution was performed using 43 brain cell-type markers across 8 categories (Supplementary Table S4) to assess detection, DAC responsiveness, and direct epigenetic target status (Supplementary Figure S4).

### 2.6. Single-Cell RNA-Sequencing Analysis

scRNA-seq data (GSE261188) were analyzed in Seurat v4 with DoubletFinder (BCmvn-optimized pK; expected doublet rate 5%). After doublet removal and QC (200 < genes < 6000; mitochondrial < 20%), 21,676 of 29,769 cells were retained (72.8% recovery; 12,841 DMSO, 8835 DAC). Normalization used SCTransform; batch correction used Harmony, which mixed both GSC models across all clusters [31,32,33,34]. Dimensionality reduction used PCA (50 PCs) and UMAP (n_neighbors = 30, min_dist = 0.1), with Louvain clustering at resolution = 0.8 (12 clusters). Cell states were assigned from Neftel et al. marker signatures via per-cell module scoring; 6 cells (0.03%) with ambiguous scores (no state passing the assignment threshold) were excluded from state-stratified analyses, leaving 21,670 cells classified across 8 states. Centroid shifts were calculated as Euclidean distances between condition-stratified cluster centroids in UMAP space. Because UMAP preserves only local topology, centroid shifts are reported as relative ranks rather than absolute distances; the IFN-response state showed the largest displacement (rank 1 of 8), with all other non-MES/stem states < 1.0. Differential abundance used Fisher’s exact test with BH correction (q < 0.05). Module score comparisons used Wilcoxon signed-rank tests with BH correction. Cell-level tests across two biological replicates (GS025, GS243) constitute pseudoreplication; single-cell results are hypothesis-generating.

### 2.7. Six-Layer Multi-Omics Integration

Six layers (L1 TMZ DEGs; L2 DAC consensus; L3 reversal candidates; L4 direct epigenetic targets; L5 single-cell state markers; L6 [25] TFs) yielded a 0–6 penetrance score per gene. Tier-1 (composite score ≥ 4; n = 11) and Tier-2 (n = 165) were combined as the deduplicated union of all ≥3-layer-penetrance genes, the 8 strictly reversed genes, and the top epi_score + TF activity genes (final 176-candidate set). To benchmark the six-layer penetrance score against random expectation, we recomputed layer penetrance after independently permuting each layer’s gene membership (10,000 permutations preserving per-layer marginal counts) and assessed candidate-set stability by leave-one-layer-out re-derivation and 2000-fold gene resampling (Supplementary Figure S7).

### 2.8. Machine Learning Validation

ML1 (logistic regression with L1 (LASSO) regularization [35]; glmnet, alpha = 1): classified DAC-treated vs. control samples; performance evaluated by AUC (5-fold cross-validation). ML2 (K-means clustering (k = 2, 100 random initializations); applied to GSVA scores): assessed by silhouette coefficient. ML3 (ridge regression; glmnet, alpha = 0): tested whether TMZ log_2_FC predicts DAC log_2_FC; measured by R^2^ with 10-fold cross-validation (mean R^2^ = 0.019, SD of per-fold R^2^ = 0.018) and 1000-permutation significance testing (perm *p* < 0.001).

ML3 cross-validation stability. The 10-fold cross-validated R^2^ estimates show substantial fold-to-fold variance: mean R^2^ = 0.019, SD = 0.018, range [−0.023, +0.042]. This instability is consistent with the small biological signal (TMZ features explain only ~2% of DAC response variance, see Supplementary Figure S6A). Across 1000 permutations of the response variable, the observed in-sample R^2^ is statistically significant (perm *p* < 0.001) due to the large gene-level sample size (n = 7489). Still, the absolute effect size is biologically negligible. This combination of statistical detectability with biologically negligible magnitude is the hallmark of a true null finding in mechanistic terms.

### 2.9. TCGA-GBM Survival Analysis

TCGA-GBM RNA-seq (HTSeq-FPKM, n = 166 primary tumors) and matched clinical data were downloaded from the UCSC Xena Browser [14] (https://xenabrowser.net/, accessed on 10 July 2026). Of the 176 prioritized candidates, 167 were present in the TCGA expression matrix. We performed univariate Cox proportional hazards regression for each gene using the survival package [36] (v3.5-5), with overall survival (OS) as the endpoint. Genes with univariate *p* < 0.05 were carried into an elastic-net Cox model (glmnet, α = 0.5; λ.min selected by 10-fold CV). This two-stage selection introduces optimism bias, so performance estimates are upper bounds (see Limitations). To quantify this optimism directly rather than assume it, we additionally performed a full-pipeline bootstrap (200 iterations) in which both the univariate *p* < 0.05 filter and the elastic-net selection were repeated within each resample. The 11-gene risk score (linear predictor) was used to stratify patients into tertiles and a median group; Kaplan–Meier curves were compared using the log-rank test, and HRs (95% CIs) were estimated from a univariate Cox model for the dichotomized groups.

### 2.10. Pharmacogenomic Mapping

*Drug*-gene interactions were identified using DGIdb v5 [15] (26 curated databases). For each of the 176 candidates, interactions with FDA-approved or investigational drugs were recorded along with interaction type and approval status. Networks were visualized using igraph and ggraph.

### 2.11. Statistical Analysis and Software

All analyses were performed in R (v4.3.0) using limma, edgeR, fgsea, Seurat, Harmony, minfi, clusterProfiler, igraph, ggraph, survival, glmnet, tidyverse, and data.table. Multiple testing was controlled using the Benjamini–Hochberg method (q < 0.05 unless otherwise specified). The FDR < 0.05 threshold was applied consistently across all analyses; where individual sections report more stringent thresholds (e.g., FDR < 0.001), these are explicitly noted. Data are presented as mean ± SD or median [IQR] depending on distribution. All tests were two-sided; *p* < 0.05 was considered statistically significant unless stated otherwise.

## 3. Results

### 3.1. An Interferon-Activated, MYC-Suppressed Transcriptional State Defines TMZ Resistance

**Figure 1:**
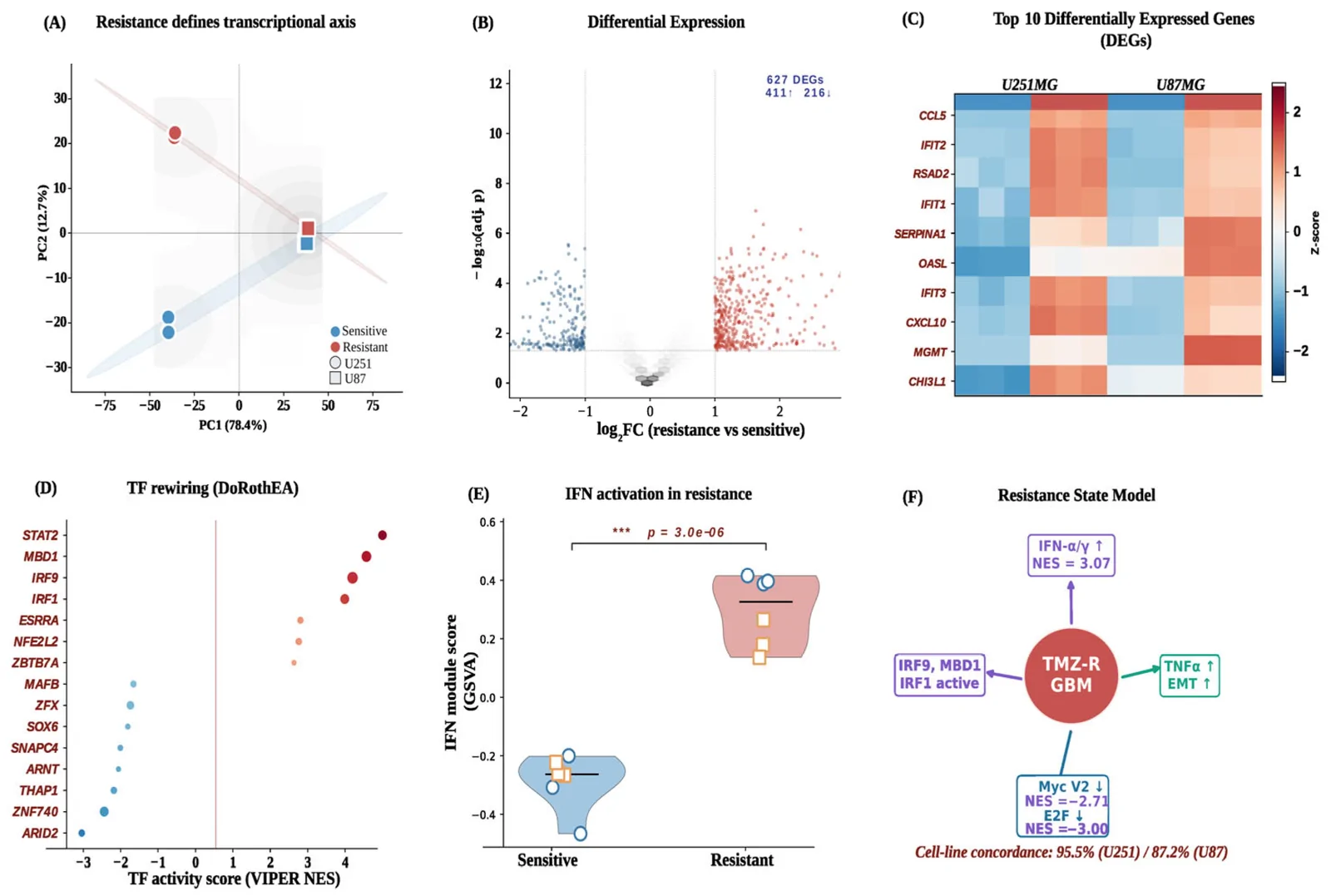
Transcriptomic landscape of TMZ resistance in GBM. (**A**) PCA of n = 12 samples (6 resistant, 6 sensitive; U251MG and U87MG); points = condition × cell-line group means, ellipses = 95% CI; PC1 captures 78.4% of variance. (**B**) Volcano of 627 DEGs (411 up, 216 down; FDR < 0.05, |log_2_FC| > 1). (**C**) Top 10 DEG heatmap (z-scores). (**D**) DoRothEA TF activity (32 active TFs; STAT2, MBD1, IRF9, IRF1 strongest). (**E**) IFN-α/γ GSVA module score (n = 6/group; Wilcoxon *p* = 3.0 × 10^−6^) and (**F**) Resistance-state schematic; cell-line-stratified signature recovery (U251MG 95.5%, U87MG 87.2%) annotated. Arrows in panel (**F**) indicate the direction of change in transcription-factor/pathway activity (↑ activated, ↓ suppressed); in panel (**E**), *** denotes *p* < 0.001 (two-sided Wilcoxon rank-sum test).

To establish the transcriptomic basis of TMZ resistance in GBM, we performed differential expression analysis on the publicly available dataset GSE151680, originally generated by Liu et al. to construct and externally validate a ferroptosis-related prognostic gene signature in glioma [17], comprising RNA-sequencing data from TMZ-resistant and TMZ-sensitive derivatives of two established GBM cell lines, U87MG and U251MG (n = 12 samples: 6 resistant, 6 sensitive). We note that this resistance signature derives from a small, two-cell-line design and is therefore hypothesis-generating, with limited generalizability to the full spectrum of GBM. Unsupervised principal component analysis (PCA) revealed a clear separation of resistant and sensitive samples along the primary axis of variance, with PC1 accounting for 78.4% of total variance (Figure 1A). Hierarchical clustering of sample-to-sample Spearman correlation matrices confirmed within-group similarity (Supplementary Figure S1).

Differential expression analysis using limma-trend (FDR < 0.05, |log_2_FC| > 1) identified 627 differentially expressed genes (DEGs; Supplementary Table S1), comprising 411 upregulated and 216 downregulated genes in resistant relative to sensitive cells (Figure 1B). DE-method robustness was confirmed by cross-method comparison of limma-trend, limma-aw, and Wilcoxon results (Supplementary Figure S1). Among the most significantly upregulated genes (Figure 1C), interferon-stimulated genes (IFIT1, IFIT2, IFIT3, RSAD2, OASL; log_2_FC ≈ 4–4.6) predominated, together with pro-inflammatory mediators CCL5 (log_2_FC = 6.61) and CXCL10, the mesenchymal markers CHI3L1 (log_2_FC = 6.18), SERPINA1, AKR1B10, and CALB2, and MGMT (log_2_FC = 5.54), the canonical TMZ-resistance determinant. Prominently downregulated genes (CXCL14, CD81, EHD2, MYEOV, E2F7; log_2_FC ≈ −3.2 to −3.9) suggest suppression of pro-apoptotic and differentiation programs in resistant cells.

We performed gene set enrichment analysis (GSEA) using the MSigDB Hallmark collection and identified 17 significantly enriched pathways (FDR < 0.05). Resistant cells were most strongly enriched for interferon alpha response (NES = 3.07, FDR < 0.001) and Interferon Gamma Response (NES = 3.05, FDR < 0.001). Other significantly enriched up-pathways included Coagulation (NES = 2.01), Inflammatory Response, TNFα signaling via NF-κB (NES = 2.79), IL-6/JAK/STAT3 signaling, and KRAS Signaling Up. Conversely, MYC Targets V1 (NES = −2.0) and MYC Targets V2 (NES = −2.71, FDR < 0.001) were strongly suppressed, alongside E2F Targets (NES = −3.00) and G2/M Checkpoint (NES = −2.70). We inferred transcription factor (TF) activity using DoRothEA, identifying 32 significantly active TFs; the strongest signals were STAT2, MBD1 (score = 4.02), IRF9 (3.65), and IRF1 (3.45) (Figure 1D), consistent with an IFN-driven transcriptional state coordinated by immune-associated regulatory hubs (DoRothEA confidence levels A-B; level C sensitivity check). GSVA scoring confirmed significantly elevated IFN-α/γ module activity in resistant relative to sensitive cells (Wilcoxon *p* = 3.0 × 10^−6^; Figure 1E). Together, these transcriptional, TF-activity, and pathway-level features define a coherent IFN-driven, Myc-suppressed mesenchymal-inflammatory resistance state, summarized schematically in Figure 1F.

### 3.2. DAC Induces Broad and Reproducible Transcriptional Reprogramming Dominated by Cancer Testis Antigen Reactivation

We next characterized the transcriptional response to DAC treatment using two independent datasets: the discovery cohort (GSE261187) and the in-domain replication cohort (GSE80137); GSE261187 (together with the companion GSE261188 and GSE261190 datasets analyzed below) was originally generated by Lai et al. to characterize DAC-induced cancer testis antigen and endogenous retrovirus reactivation in glioma at single-cell resolution [16]. Differential expression analysis with limma-trend (FDR < 0.05, |log_2_FC| > 1) identified 1114 DEGs in the discovery dataset (952 upregulated, 162 downregulated) and 1882 DEGs in the validation dataset (1832 upregulated, 50 downregulated) following DAC treatment relative to DMSO control (Supplementary Figure S2A). The strong bias toward upregulation in both datasets is consistent with DAC-mediated demethylation releasing silenced loci from transcriptional repression (Supplementary Figure S2A).

**Figure 2:**
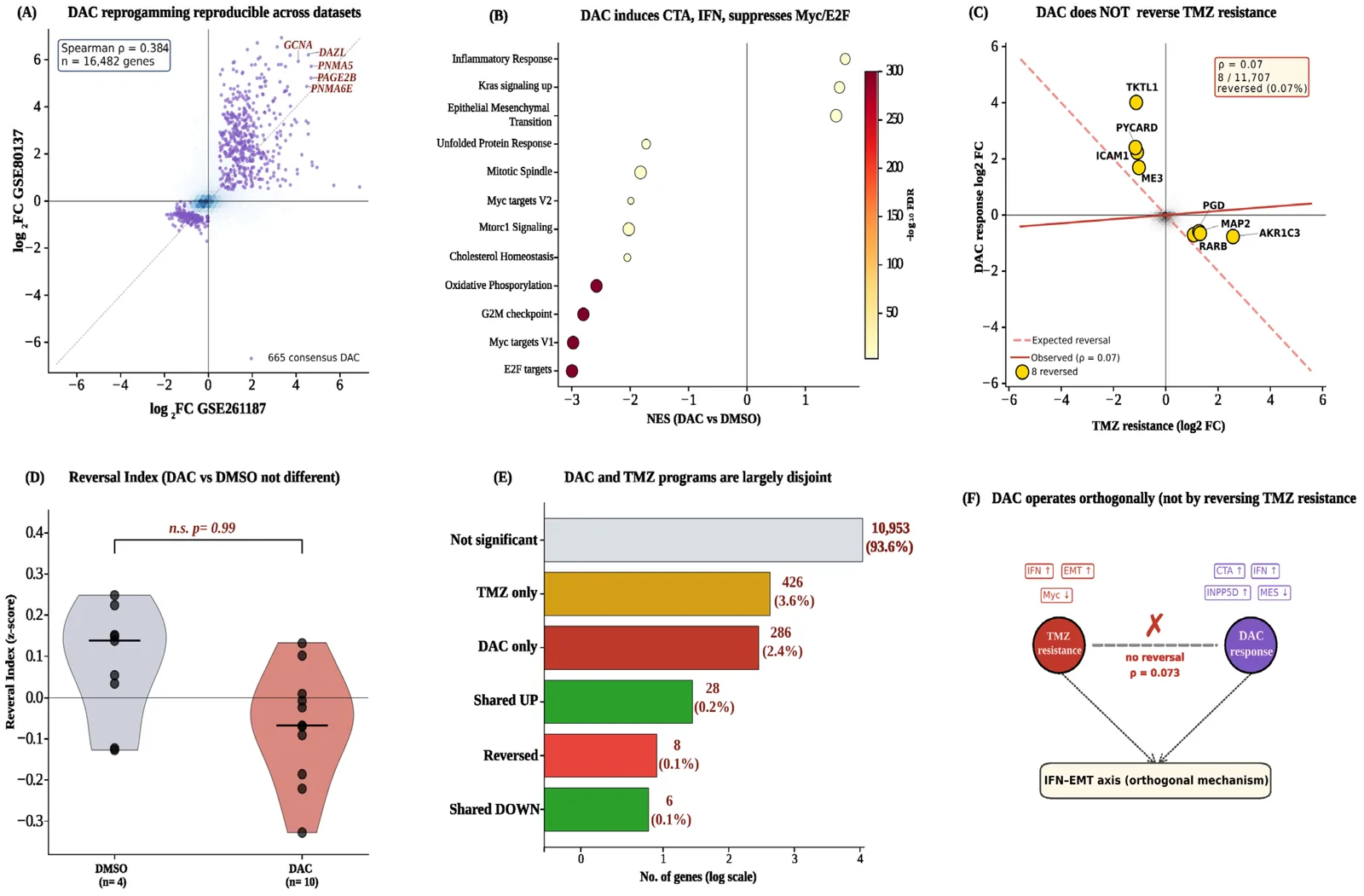
Decitabine reprograms transcription orthogonally to TMZ resistance, not by reversal. (**A**) Cross-dataset DAC reproducibility scatter (discovery × replication log_2_FC; Spearman ρ = 0.38 on 16,482 genes). (**B**) GSEA Hallmark dot plot of 665 consensus DAC-responsive genes (DAC induces CTA, IFN; suppresses Myc/E2F). (**C**) TMZ log_2_FC vs. DAC meta-effect size scatter (ρ = 0.073, shown rounded to ρ = 0.07 in the panel inset; only 8/11,707 [0.07%] strictly reversed). (**D**) Per-sample Reversal Index violin (DMSO vs. DAC; n.s., *p* = 0.99). (**E**) Gene-class bar chart partitioning the 11,707 co-detected genes (10,953 not significant; 426 TMZ-only; 286 DAC-only; 28 shared up; 8 reversed; 6 shared down) and (**F**) schematic: DAC operates orthogonally to TMZ resistance via the IFN–EMT axis rather than by reversing the resistance signature. In panel (**F**), the arrow denotes the tested DAC-versus-TMZ comparison and the red ✗ indicates the absence of significant transcriptional reversal (Spearman ρ = 0.073; *p* = 0.99).

We assessed cross-dataset concordance and observed a significant positive correlation in log_2_FC values between the discovery and in-domain replication cohorts (Spearman ρ = 0.384 (Pearson r = 0.425), *p* < 2.2 × 10^−16^, n = 16,482, Figure 2A), confirming reproducibility of the DAC-induced transcriptional program across independent experimental systems. A meta-analysis integrating both datasets yielded 665 consensus DAC-responsive genes (Supplementary Table S2; Supplementary Figure S2B). Among the most consistently upregulated genes across both datasets were cancer testis antigens (CTAs) and germ cell-specific factors, including HORMAD1, MAGEA4, PNMA6E, PNMA5, PAGE2B, DCAF4L1, DAZL, and GCNA. This coordinated CTA reactivation corroborates, in an independent, integrative bulk-RNA-seq analysis, the single-cell CTA/ERV reactivation phenotype originally reported in these same DAC-treatment datasets by Lai et al. [16] and extends prior evidence of DAC-induced immunogenic reprogramming in the GBM field [9,10,12]. GSEA of the 665 consensus DAC-responsive genes identified 23 significantly enriched Hallmark pathways (FDR < 0.05; Figure 2B), with upregulation of Inflammatory Response, EMT, TNFα/NF-κB, and IL6/JAK/STAT3 (NES = 2.09–2.54) and downregulation of E2F Targets, MYC Targets, and mTORC1 signaling (NES = −2.31 to −2.67) (Figure 2B).

### 3.3. DAC Does Not Broadly Reverse the TMZ Resistance Transcriptome: A Formal Quantitative Analysis

A recurring hypothesis in the field is that demethylating agents might directly reverse the transcriptional program driving resistance. To formally test this, we performed a systematic reversal analysis integrating the TMZ resistance signature with the DAC transcriptional response across 11,707 genes consistently detected in both GSE151680 and the DAC response datasets. Across all 11,707 genes, the Spearman rank correlation between TMZ log_2_FC values and DAC effect sizes was negligible (ρ = 0.073; Pearson r = 0.114), indicating no evidence of genome-wide transcriptional reversal. We defined “strict reversal” as genes upregulated in TMZ resistance and downregulated by DAC (or vice versa) under stringent criteria (|log_2_FC| > 1 in TMZ; FDR < 0.05 in both datasets; opposite directions of effect; Supplementary Figure S2C,D). Eight genes met these strict reversal criteria (0.07% of all analyzed genes): AKR1C3 (TMZ log_2_FC = 2.57, DAC log_2_FC = −0.77), MAP2 (1.27, −0.59), ME3 (−1.03, 1.69), ICAM1 (−1.10, 2.24), PYCARD (−1.16, 2.41), PGD (1.07, −0.70), and TKTL1 (−1.13, 4.01) were carried forward into the 176-candidate set; RARB (1.31, −0.66) was excluded for failing the integration threshold (Figure 2C).

To quantify reversal more formally, we calculated a Reversal Index (RI) across six GBM cell line models (GS025, GS081, GS243, GS277, GS351, and U251). Observed RI values were compared to a sample-label-permutation null distribution (10,000 permutations) using a Wilcoxon signed-rank test, which showed no significant enrichment (Figure 2D; Supplementary Figure S2E; *p* = 0.990; n = 4 DMSO and n = 10 DAC samples after per-model paired-sample QC). The reversal candidate’s signature was the sole predefined gene signature not to reach the pre-specified α = 0.05 threshold in in-domain replication (GSE80137; 7/7 genes modulated in the expected direction, Δ = +1.81, *p* = 0.065).

Across-class classification of the 11,707 co-detected genes yielded six mutually exclusive groups (Figure 2E): 10,953 not significant; 426 TMZ-specific; 286 DAC-specific; 34 concordant genes (28 shared up + 6 shared down); and 8 strictly reversed (Fisher’s exact one-sided *p* = 5.8 × 10^−5^ for the 34 shared vs. expected 16.6; hypergeometric one-sided *p* = 5.8 × 10^−5^). The shared-gene class reflects co-activation of inflammatory and immune-related programs despite overall mechanistic divergence. Together, these results support a model in which DAC operates orthogonally to TMZ resistance via the IFN–EMT axis rather than by direct transcriptional reversal (Figure 2F).

### 3.4. Genome-Wide DNA Methylation Profiling Identifies 146 Direct Epigenetic Targets, Including INPP5D/PI3K as a Putative Regulatory Target

**Figure 3:**
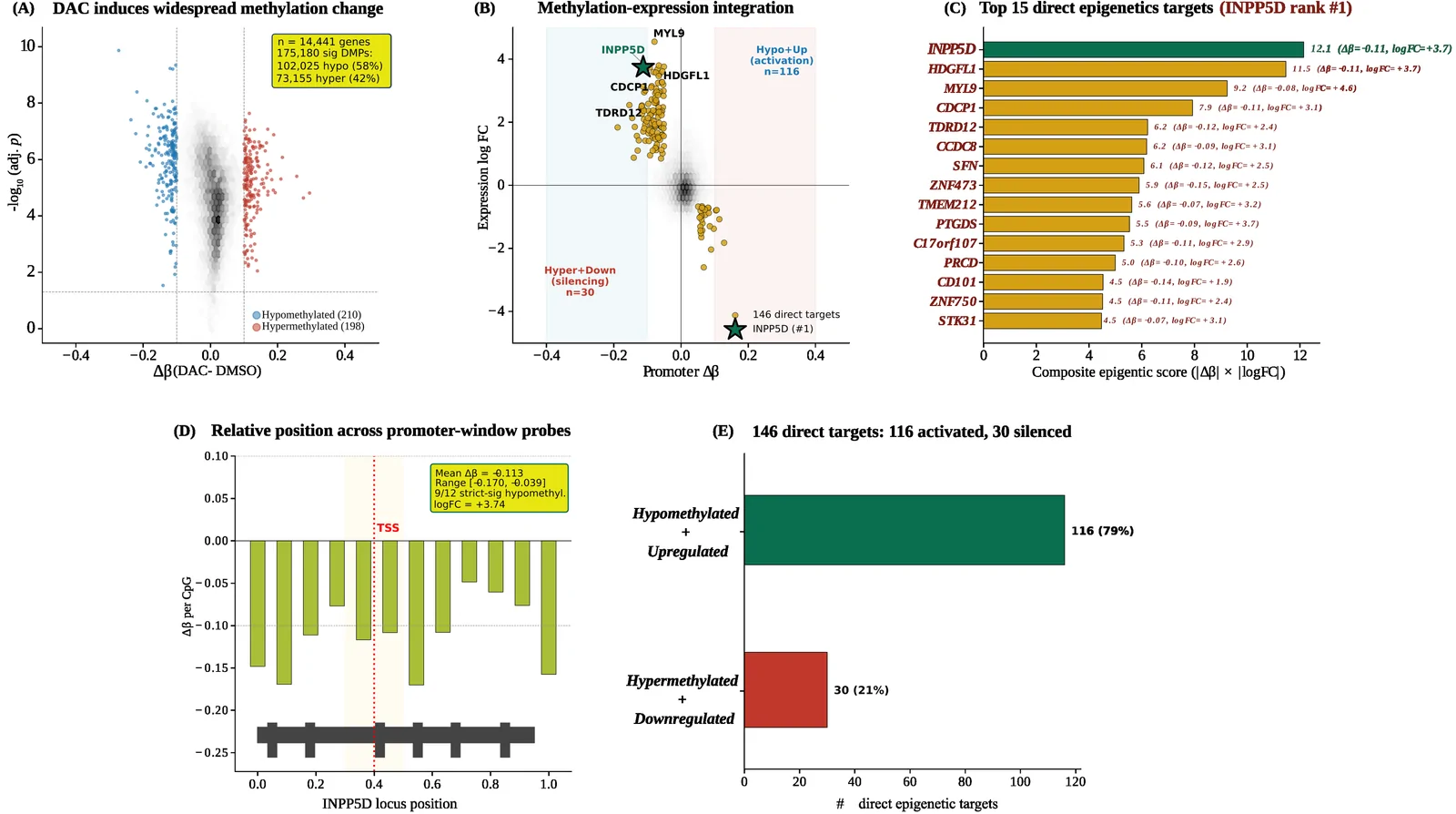
Methylation–expression integration and direct epigenetic targets of DAC. (**A**) Differentially methylated position (DMP) volcano plot across 803,785 quality-filtered EPIC probes mapped to 14,441 unique genes (see Section 2.5). Under the stringent threshold (|Δβ| > 0.10 and BH-adjusted *p* < 0.05), 175,180 CpGs are significant (102,025 hypomethylated, 73,155 hypermethylated). The hypomethylated (210) and hypermethylated (198) labels in the bottom legend denote the thresholded subsets of representative DMPs visualized in the plot for clarity (not genome-wide totals). (**B**) Methylation × expression integration scatter (INPP5D rank #1; Hypo + Up = activation, n = 116; Hyper + Down = silencing, n = 30). (**C**) Top 15 direct epigenetic activation targets ranked by composite epi-score (|Δβ| × |log_2_FC|); INPP5D, HDGFL1, MYL9, CDCP1, TDRD12 lead. (**D**) INPP5D locus: paired Δβ across 12 CpG probes spanning the gene model (mean Δβ = −0.113; 9/12 strict-significant hypomethylation; logFC = +3.74) and (**E**) Integration-class composition: of 146 direct epigenetic targets, 116 (79%) were Hypo + Up (activated) and 30 (21%) were Hyper + Down (silenced). In panels (**B**,**C**), “#” denotes rank position by composite epigenetic score (e.g., INPP5D rank #1 = top-ranked). In panel (**E**), green denotes hypomethylated + up-regulated (direct activation) targets and red denotes hypermethylated + down-regulated (direct silencing) targets.

To examine how DAC-induced transcriptional changes relate to DNA methylation, we integrated EPIC 850K methylation data (GSE261190; 803,785 probes retained after quality filtering; Supplementary Figure S3A) with gene expression profiles. After mapping to gene annotations (±2 kb from the TSS for promoters, plus gene body, UTR, and enhancer regions), the 803,785 retained CpGs mapped to 14,441 unique genes, which constitute the methylation–expression integration background. Differential methylation analysis using limma on M-values (|Δβ| > 0.1 and BH-adjusted *p* < 0.05) identified 175,180 significantly altered CpGs: 102,025 hypomethylated (Δβ < −0.1) and 73,155 hypermethylated (Δβ > 0.1; Figure 3A; Supplementary Figure S3B). Δβ distributions were broadly similar across genomic regions (promoter, gene body, enhancer, UTR, intergenic; Supplementary Figure S3C); region-stratified DMP volcano in Supplementary Figure S3E. This bidirectional pattern is expected given DAC’s predominant demethylating activity, with the locus-specific hypermethylation likely reflecting secondary effects on de novo methylation machinery. The integration-class composition (methylation-only, expression-only, concordant Hypo + Up, concordant Hyper + Down) is summarized in Supplementary Figure S3F. Integration of promoter-level methylation changes with gene expression changes identified 146 direct epigenetic targets (Figure 3B; Supplementary Table S3): 116 genes with promoter hypomethylation coupled to upregulation (direct activation targets) and 30 genes with promoter hypermethylation coupled to downregulation (direct silencing targets). We ranked genes by a composite epigenetic score (epi_score = |Δβ| × |log_2_FC|). Among the top direct activation targets, INPP5D ranked first (epi_score = 12.14), HDGFL1 (11.47), MYL9 (9.24), CDCP1 (7.93), and TDRD12 (6.22) (Figure 3C). Direct silencing targets included FABP7, ALPK3, LIPI, RGMA, and S100A4. The INPP5D locus showed concordant Δβ across 12 CpG probes spanning the gene model (mean Δβ = −0.113; 9/12 strictly significant hypomethylation; logFC = +3.74; Figure 3D), and the overall integration-class composition (116 Hypo + Up activation targets, 30 Hyper + Down silencing targets) is summarized in Figure 3E.

Given the clinical relevance of MGMT promoter methylation, all 191 EPIC probes annotated to MGMT were examined (Supplementary Figure S3D); the 22-probe canonical CpG-island promoter showed only a minor Δβ of −0.025 (95% CI −0.05–0.00), with no probe meeting strict significance and a baseline DMSO β of 0.31 indicating a partially open promoter consistent with a floor effect. Six significant intronic probes lie outside the regulatory promoter. INPP5D encodes SHIP1, a negative regulator of PI3K/AKT signaling. Because INPP5D (SHIP1) is predominantly expressed in microglia and brain macrophages, we performed an in silico marker-based deconvolution to exclude residual contamination of the GSC cultures (Supplementary Table S4 and Supplementary Figure S4). Canonical microglial markers (CX3CR1, CSF1R, AIF1, TREM2, TYROBP) were below detection, and INPP5D’s elevated baseline methylation (β_DMSO = 0.733) is inconsistent with constitutive microglia, supporting tumor-cell-autonomous silencing. Given the prevalence of PI3K/AKT hyperactivation in GBM, DAC-mediated INPP5D reactivation may attenuate PI3K signaling independently of DAC’s immune-activating effects. TDRD12 reactivation is consistent with coordinated demethylation of germline loci. Among direct silencing targets, FABP7 and S100A4 are established drivers of mesenchymal/invasive GBM identity; paradoxical DAC-induced hypermethylation at S100A4 implicates indirect chromatin effects that may contribute to the MES-like attenuation seen in single-cell data.

### 3.5. Single-Cell Analysis Reveals DAC-Induced Cell State Modulation and Composition Shifts

**Figure 4:**
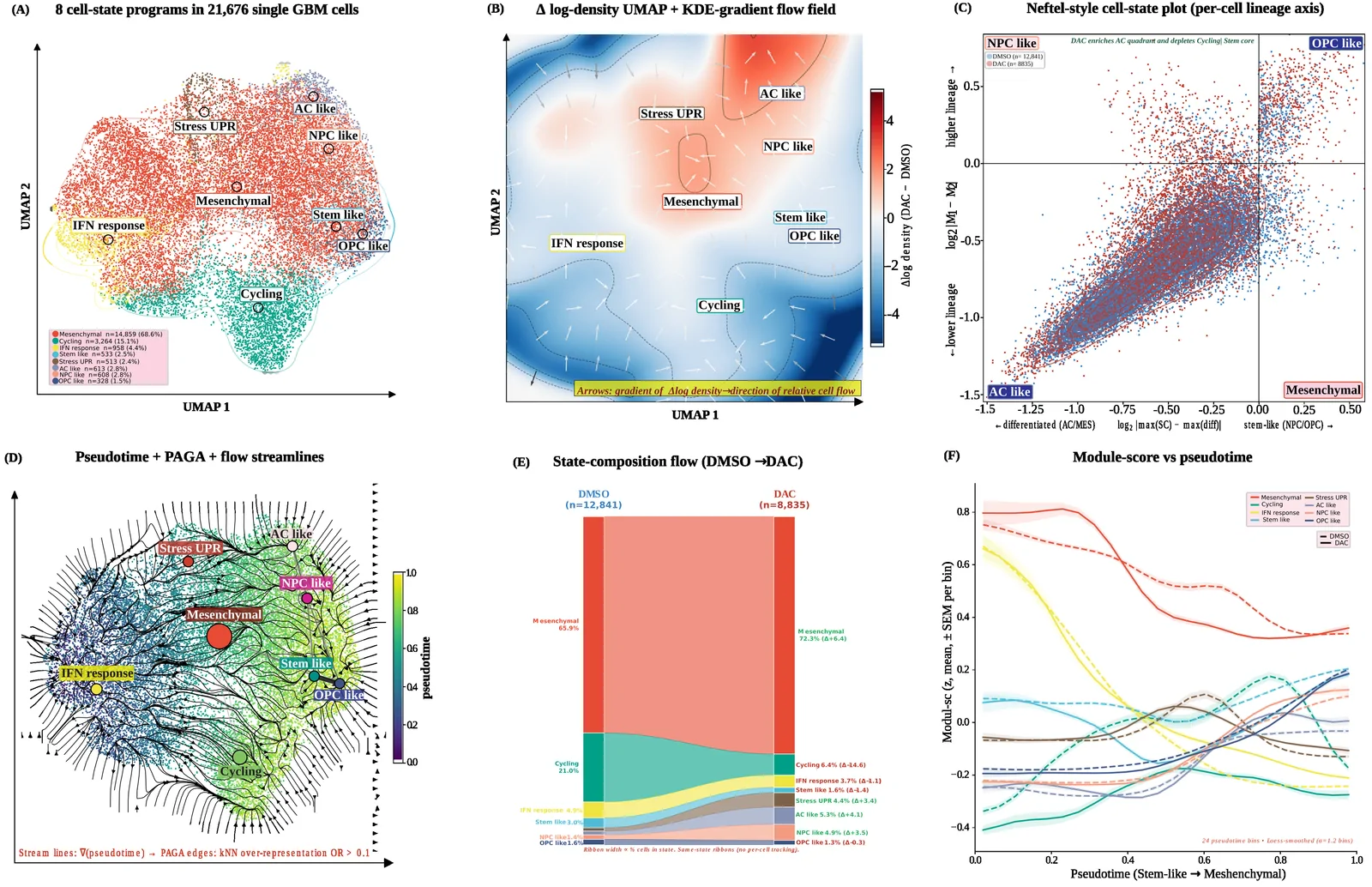
Single-cell analysis of DAC-induced cell-state modulation across 21,676 cells. (**A**) UMAP of 21,676 cells colored by 8 cell-state programs. (**B**) Δ log-density UMAP (DAC − DMSO) with KDE flow-field arrows showing direction of relative cell-density change. (**C**) Neftel-style per-cell lineage plot (AC-like/mesenchymal/NPC-like/OPC-like axes); DAC enriches the AC-like quadrant and depletes the cycling/stem-like core. (**D**) Pseudotime + PAGA backbone with flow-streamline overlay; pseudotime color-encoded (purple = early, yellow = late), trajectory rooted at cycling. (**E**) State-composition Sankey from DMSO (n = 12,841) to DAC (n = 8835); ribbon width = % cells and (**F**) Loess-smoothed module-score (z-mean ± SEM) vs. pseudotime across 24 bins for all 8 states, DMSO (solid) vs. DAC (dashed).

To characterize cellular heterogeneity and assess state-specific DAC effects at single-cell resolution, we analyzed the GSE261188 dataset comprising 21,676 high-quality cells (12,841 DMSO-treated and 8835 DAC-treated; 72.8% recovery rate from 29,769 pre-QC cells; per-sample mitochondrial percentage distributions in Supplementary Figure S5A) across two glioblastoma stem-cell (GSC) models, GS025 (classical, IDH-wildtype) and GS243 (mesenchymal, IDH-wildtype), as annotated in the GSE261188 source publications [16], most directly by Lai et al., who generated and originally characterized this dataset [16]. After SCTransform normalization, Harmony batch correction, and Louvain clustering (resolution = 0.8), 12 clusters were identified and annotated into 8 biologically distinct cell states based on Neftel et al. marker gene signatures: MES-like (68.5%, n = 14,855), cycling (15.1%, n = 3264), IFN-response (4.4%, n = 956), AC-like (2.8%, n = 613), NPC-like (2.8%, n = 608), stem-like (2.5%, n = 533), stress/UPR (2.4%, n = 513), and OPC-like (1.5%, n = 328); the remaining 6 cells (0.03%) had ambiguous Neftel-marker scores and were excluded from state-stratified module-score comparisons (Figure 4A; Supplementary Figure S5B–D).

Upon stratification by treatment, UMAP visualization revealed that DAC induced substantial shifts in cell-state occupancy (Figure 4B). Centroid shift analysis quantified the Euclidean displacement in 2-D UMAP space between DMSO and DAC conditions for each state. This analysis revealed the IFN-response state underwent the largest displacement (d = 3.84), followed by MES-like (d = 2.75) and stem-like (d = 1.86) states (Figure 4C). Centroid shifts (UMAP topology) and module-score Δ (within-cell intensity) measure orthogonal properties. DAC significantly reduced stem-like scores (Δ = −0.135, *p* < 0.001), cycling scores (Δ = −0.273, *p* < 0.001), and MES-like scores (Δ = −0.071, *p* < 0.001), while the stress/UPR module score showed a significant increase (Δ = +0.041, *p* < 0.001; Wilcoxon signed-rank test, Benjamini–Hochberg adjusted; Supplementary Figure S5E). These data indicate that DAC reduces per-cell mesenchymal and stem-like transcriptional intensity in populations linked to therapeutic resistance in GBM. However, as detailed in Section 3.6, this per-cell attenuation must be distinguished from changes in population-level proportions. These two observations are complementary rather than contradictory: DAC lowers the mesenchymal transcriptional intensity within individual cells, while the MES-like population fraction rises only passively, through preferential depletion of cycling cells. Population-level analysis showed concordant DAC-induced shifts across both GSC models (Figure 4E): the MES-like fraction rose from 65.9% to 72.3% (Δ = +6.4%), a paradoxical increase driven not by mesenchymal expansion but by preferential depletion of cycling cells (−14.6%), with smaller decreases in IFN-response (−1.1%) and stem-like (−1.4%) populations and compensatory increases in AC-like (+4.1%), NPC-like (+3.5%), and stress/UPR (+3.4%) compartments (OPC-like Δ = −0.3%) (Fisher’s exact, BH-adj. *p* < 0.01; Figure 4E). Cluster marker analysis confirmed proliferation genes (KIF20A, NEK2, PTTG1, MCM10, RAD51) in cycling clusters and neuronal/astrocytic markers (CACNG8, ELAVL4, VSNL1, RGS4) in differentiated-state clusters (Supplementary Figure S5D). Because these single cells derive from only two GSC models and are not independent biological replicates, all cell-level *p*-values overstate statistical significance and are interpreted here as hypothesis-generating only.

Per-cell lineage analysis on Neftel axes (Figure 4C) showed that DAC enriched the AC-like quadrant and depleted the cycling/stem-like core, while preserving the mesenchymal-versus-NPC/OPC lineage spread. Pseudotime trajectory inference with PAGA backbone reconstruction (Figure 4D) anchored the trajectory at the cycling cluster as the inferred root, with MES-like and stem-like clusters occupying adjacent intermediate positions and IFN-response cells branching off as a distinct trajectory endpoint. Module-score-versus-pseudotime curves for all 8 cell states (Figure 4F) showed that DAC consistently shifted MES-like and IFN-response trajectories downward across pseudotime, while stress/UPR scores rose in late-pseudotime cells, supporting the per-cell attenuation observed in the module-score analysis above.

### 3.6. Six-Layer Multi-Omics Integration Identifies 176 Prioritized Candidates and Reveals Convergent Interferon-EMT Biology

**Figure 5:**
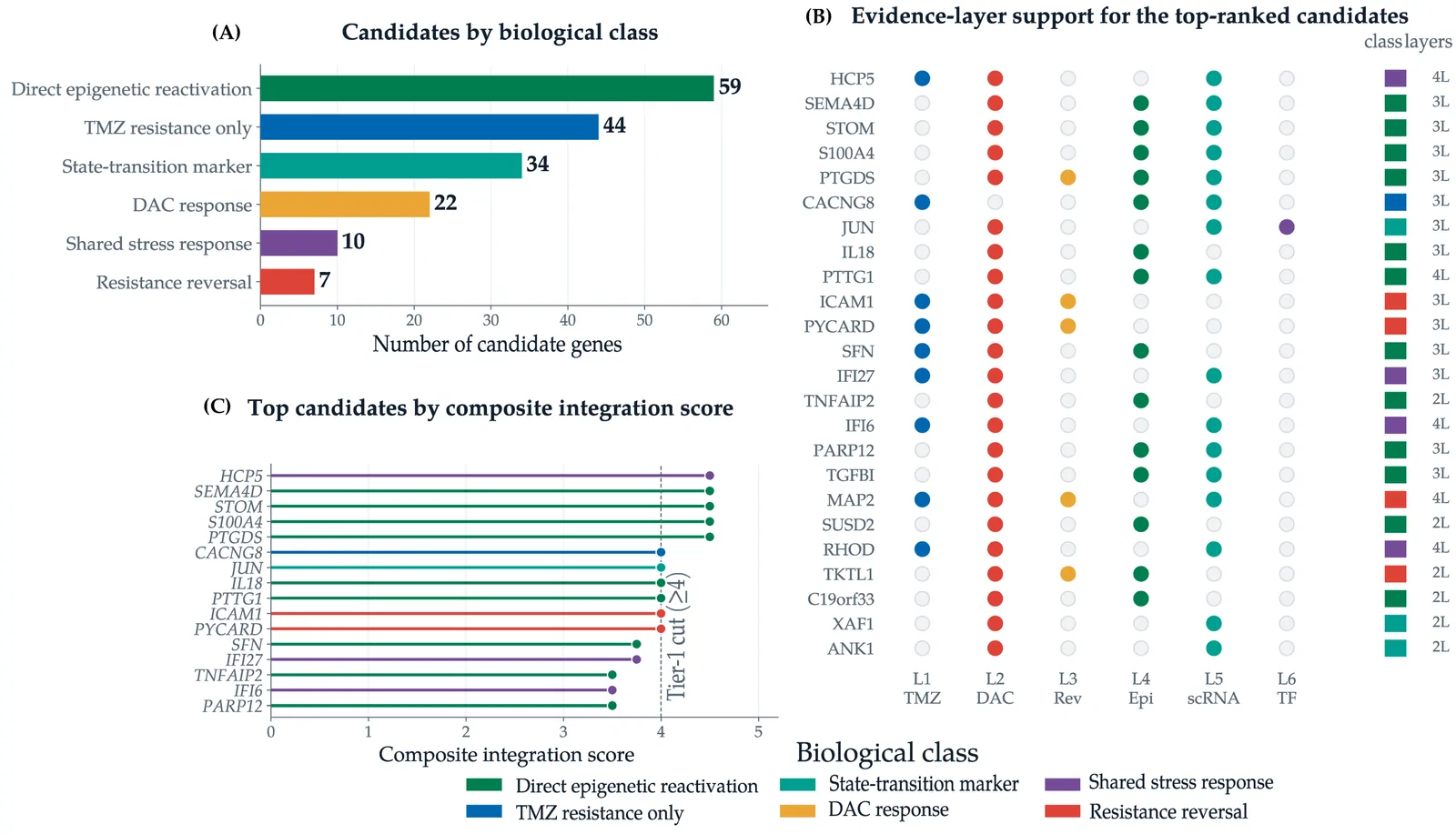
Six-layer integration prioritizes 176 candidates converging on an interferon–EMT axis. (**A**) Number of prioritized candidate genes in each of the six biological classes (direct epigenetic reactivation, n = 59; TMZ resistance only, n = 44; State-transition marker, n = 34; DAC response, n = 22; Shared stress response, n = 10; Resistance reversal, n = 7). (**B**) Evidence-layer support for the 24 top-ranked candidates: a filled circle indicates that the gene is supported by layer L1 (TMZ-resistance DEG), L2 (DAC consensus response), L3 (reversal/shared), L4 (direct epigenetic target), L5 (single-cell state marker) or L6 (active transcription factor); the colored tag at right denotes biological class and the adjacent value gives the number of contributing layers. (**C**) Top candidates ranked by composite integration score; the dashed line marks the Tier-1 threshold (composite score ≥ 4). Colors denote biological class throughout (key, bottom).

We developed a six-layer evidence scoring system: L1 TMZ resistance DEGs (n = 627), L2 DAC consensus genes (n = 665), L3 reversal candidates (8 strictly reversed + 34 shared), L4 direct epigenetic targets (n = 146), L5 single-cell state markers, and L6 DoRothEA-inferred active TFs (n = 32), and computed a 0–6 layer-penetrance score across 9708 genes, with the integrated penetrance rings and the six biological-class arcs shown together in Figure 5A. Applying a pre-specified three-criterion selection rule (≥3-layer penetrance, the 8 strict-reversal genes, and top-ranked composite epi-score + TF activity; Section 2.7), we obtained 176 unique prioritized candidates partitioned across six biological classes: direct epigenetic reactivation (n = 59), TMZ resistance only (n = 44), state-transition marker (n = 34), DAC response (n = 22), Shared stress response (n = 10), and resistance reversal (n = 7; RARB excluded), yielding Tier-1 = 11 (composite score ≥ 4) and Tier-2 = 165 (Figure 5A; Supplementary Table S5). Class proportions and the stepwise decomposition of candidates by integration class (Hypo + Up 47, Hyper + Down 12, Methylation Only 3, Expression Only 100, NS 14) and TF-network status (In Network 74, Not in Network 102) are visualized in Figure 5C. To test whether this multi-layer convergence exceeds random expectation, we compared observed penetrance with a label-permutation null (10,000 permutations preserving per-layer marginals). Genes supported by ≥4 of the six layers (n = 12, closely matching the 11 Tier-1 candidates) were strongly non-random (0.7 ± 0.8 expected; ~18-fold enrichment; empirical *p* < 1 × 10^−4^), whereas the broader ≥ 3-layer set was only modestly above chance (50 observed vs. 44 ± 6 expected; *p* = 0.19), reflecting the breadth of the single-cell state-marker layer, which annotates ~75% of genes. Leave-one-layer-out analysis showed the prioritized set depended most on the DAC-consensus layer (Jaccard 0.26 on removal) and least on the transcription-factor layer (0.96), and the ≥3-layer count was stable under gene resampling (bootstrap 95% CI 37–64). The 176-candidate list is therefore best interpreted as a hypothesis-generating superset, with the Tier-1 convergent core as its statistically robust subset (Supplementary Figure S7).

Per-gene evidence profile analysis identified nine top-scoring candidates (composite score = 3.0; Figure 5C): HCP5, PTTG1, MAP2, CACNG8, SEMA4D, S100A4, STOM, JUN, and PTGDS, with HCP5 showing the broadest multi-layer support (L1 TMZ DEG, L2 DAC consensus, L3 shared, and L6 active TF). JUN integrated three layers of evidence (L2 DAC consensus, L5 single-cell state-transition marker, and L6 active TF), consistent with an immediate-early/AP-1 role that is active in both the DAC response and mesenchymal-state transitions. CACNG8 also reached three-layer penetrance (L1: TMZ resistance, L4: direct epigenetic target, and L5: single-cell state marker), reflecting concordant evidence of TMZ resistance, methylation, and state transition.

In-domain replication in GSE80137 confirmed significant DAC modulation of four of five predefined signatures: DAC epigenetic reactivation (19/20; Δ = +2.473), DAC Response Core (30/30; Δ = +1.248), state transition (20/20; Δ = +0.960), and TMZ Resistance Core (29/30; Δ = +0.663) at *p* ≤ 8.7 × 10^−3^, while only the reversal candidates signature failed (7/7; Δ = +1.81; *p* = 0.065), reinforcing that direct transcriptional reversal is not the primary mechanism of DAC. Overlap with published GBM signatures was highest for IFN/viral mimicry (50%) and GBM mesenchymal (40%), anchoring a convergent interferon–EMT axis. GENIE3 inference yielded a 220-node/1052-edge full network; the 74-node/796-edge TF-regulatory subgraph (Figure 5B) is centered on JUN as the principal hub (out-degree = 29; Supplementary Table S6).

### 3.7. Machine Learning Analysis Supports Mechanistic Independence of TMZ and DAC Transcriptional Programs

To test whether the TMZ-resistance and DAC response programs are mechanistically independent, we applied three machine-learning approaches at sample, gene-set, and gene-expression-prediction levels (Supplementary Table S7). LASSO logistic regression cleanly separated DAC- from control samples (AUC = 0.776), driven by interferon-stimulated genes and inflammatory markers (IFIT1, IFIT2, ISG15, IL6). Ridge regression predicting DAC log_2_FC from TMZ log_2_FC yielded a 10-fold cross-validated R^2^ of 0.019 (SD = 0.018; range −0.023 to +0.042; Supplementary Figure S6A), confirming that the two programs are largely independent.

### 3.8. Exploratory TCGA-GBM Survival Analysis in 166 Patients (Hypothesis-Generating Prognostic Model)

To evaluate the clinical relevance of the prioritized candidates in an exploratory, hypothesis-generating analysis, we performed an internal cross-validated survival analysis using TCGA-GBM RNA-sequencing expression data and clinical information from 166 patients (median follow-up: 360 days, IQR: 158–534 days; 133 events). Of 176 candidate genes, 167 were found in the TCGA-GBM expression dataset. Univariate Cox proportional hazards regression was applied to these genes using standardized expression values (z-scores; Supplementary Table S7).

**Figure 6:**
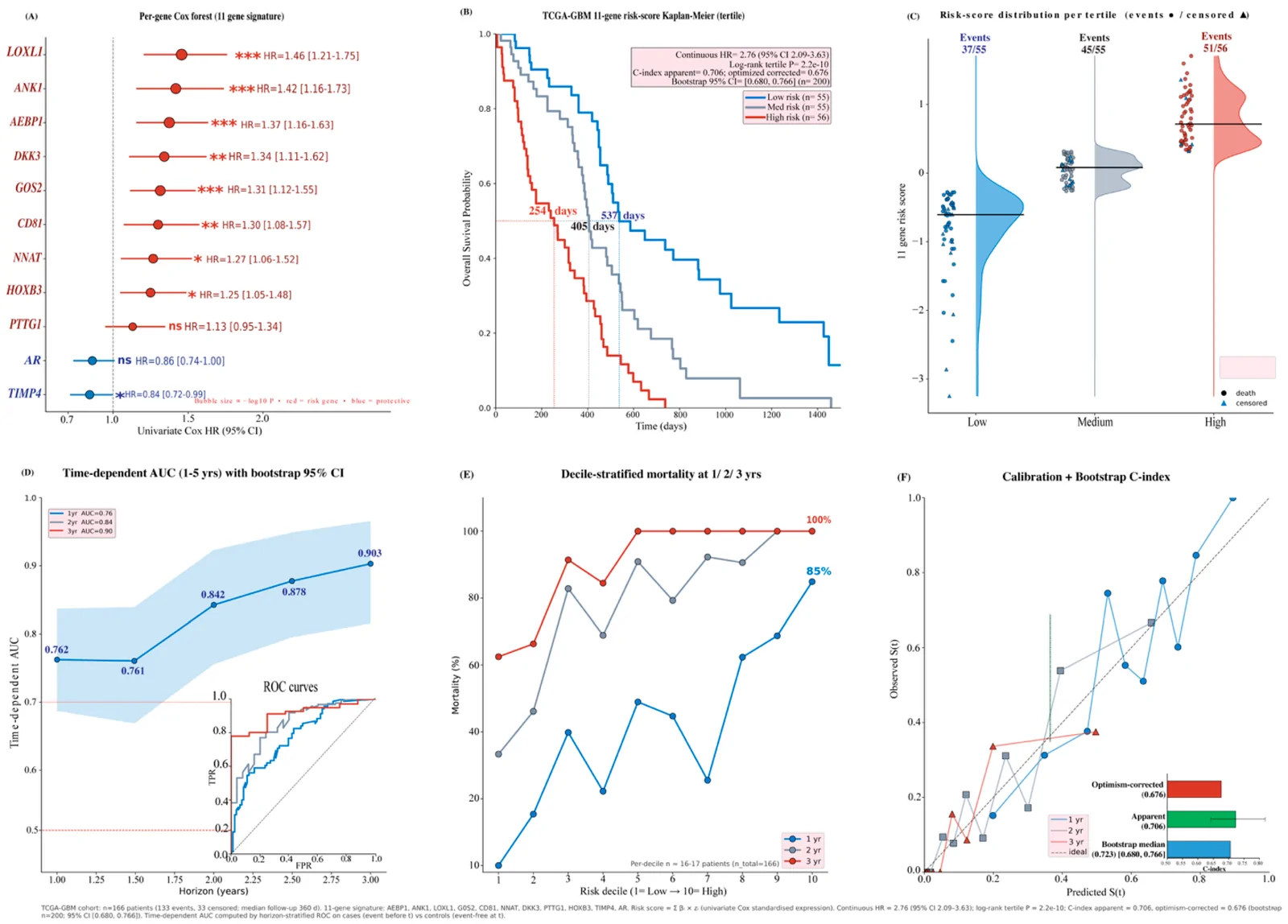
TCGA-GBM validation of the 11-gene prognostic signature. (**A**) Per-gene univariate Cox forest plot for the 11-gene signature (HRs and 95% CIs in Supplementary Table S7); bubble size = −log_10_ *p*; red = risk, blue = protective. (**B**) Kaplan–Meier OS by tertile (Low n = 55, Med 55, High 56; log-rank *p* = 2.2 × 10^−10^; median OS 537/405/254 days). Inset: continuous HR = 2.76 (95% CI 2.09–3.63); apparent C-index = 0.706, optimism-corrected = 0.676. (**C**) Risk-score distribution per tertile (event vs. censored). (**D**) Time-dependent ROC AUC at 1–5 years with bootstrap 95% CI (n = 166; 133 events). (**E**) Decile-stratified mortality at 1, 2, 3 years (per-decile n ≈ 16–17) and (**F**) calibration plot at 1, 2, 3 years; inset bars: apparent 0.706, optimism-corrected 0.676, bootstrap median 0.723. Significance annotations: * *p* < 0.05, ** *p* < 0.01, *** *p* < 0.001, ns = not significant.

Univariate Cox regression identified 31 genes significantly associated with overall survival (OS) at *p* < 0.05, with 4 genes—LOXL1, AEBP1, ANK1, and G0S2—remaining significant after Benjamini–Hochberg correction (adj. *p* < 0.05) (Figure 6A). Effect sizes were LOXL1 (HR = 1.46, *p* = 0.0001), AEBP1 (HR = 1.37, *p* = 0.0002), ANK1 (HR = 1.42, *p* = 0.0007), and G0S2 (HR = 1.31); SOX6 was the strongest protective gene at the nominal threshold (HR = 0.79). An 11-gene elastic net Cox regression model was constructed incorporating AEBP1, ANK1, LOXL1, G0S2, CD81, NNAT, DKK3, PTTG1, HOXB3, TIMP4 (protective), and AR (protective; sex-stratified analysis was not performed, and AR’s protective direction may partially reflect sex composition of the TCGA-GBM cohort). These genes were selected from univariate-significant candidates and validated biological pathway markers; per-gene expression density across the 166 TCGA-GBM samples is shown in Supplementary Figure S6B, and pairwise gene–gene correlations are shown in Supplementary Figure S6C. Patients stratified by the 11-gene risk model showed separation in survival (Figure 6B). The model achieved an apparent C-index of 0.706 and an optimism-corrected C-index of 0.676 (optimism = 0.029), indicating moderate but honest discrimination. When the two-stage gene selection was itself repeated inside a full-pipeline bootstrap, optimism increased to 0.062, giving a selection-inclusive optimism-corrected C-index of 0.63 (apparent-index bootstrap 95% CI 0.66–0.75; permutation *p* = 0.0002 versus a random-risk null). The model is therefore exploratory and hypothesis-generating; external validation in the independent CPTAC-GBM cohort (n = 96, 62 deaths; all 11 genes measured) showed only near-chance discrimination (C-index = 0.55, 95% CI 0.47–0.63; high- vs. low-risk tertile log-rank *p* = 0.33; HR = 1.27 per SD, 95% CI 0.99–1.63), indicating that the signature does not generalize beyond TCGA-GBM and should be regarded strictly as an exploratory, hypothesis-generating tool rather than a prognostic biomarker (Supplementary Figure S8; see Limitations). The continuous risk score yielded HR = 2.76 per unit (95% CI 2.09–3.63); tertile-stratified high-vs-low yielded HR = 4.59 (95% CI 2.85–7.40, log-rank *p* = 2.2 × 10^−10^; median OS = 537/405/254 days for Low/Med/High tertiles, Figure 6B); risk-score distribution per tertile shown in Figure 6C. Time-dependent ROC AUC at 1–5 years was estimated by 200-iteration bootstrap (Figure 6D), yielding 1-year AUC = 0.76, 2-year AUC = 0.84, and 3-year AUC = 0.90 with overlapping bootstrap 95% CIs, and decile-stratified mortality at 1/2/3 years is shown in Figure 6E. Schoenfeld residuals confirmed the proportional-hazards assumption at α = 0.05 in the overall Cox model (Supplementary Figure S6D; Supplementary Table S7). Calibration at 1, 2, and 3 years is shown in Figure 6F. Censoring decreased monotonically across risk strata (Low 32.7%, Med 18.2%, High 8.9%; Supplementary Table S7), so the high-risk arm had near-complete event observation.

### 3.9. Pharmacogenomic Landscape and Therapeutic Opportunities

**Figure 7:**
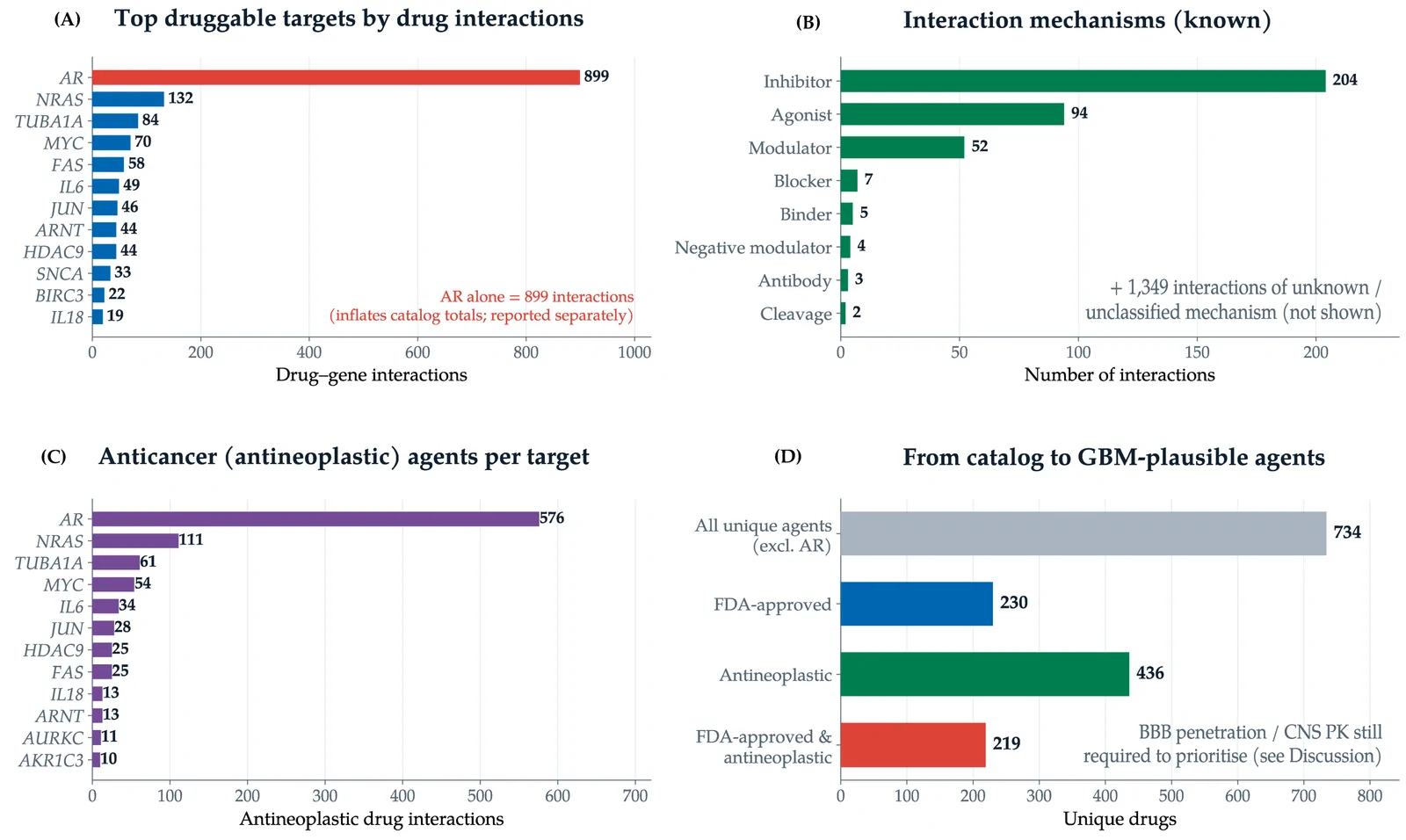
Pharmacogenomic landscape of the 176 candidates (DGIdb v5), presented as a catalog-level, hypothesis-generating resource. (**A**) Top druggable targets by number of drug–gene interactions; AR is an outlier (899 interactions) and is reported separately. (**B**) Known interaction mechanisms (a further 1349 interactions have unknown/unclassified mechanisms). (**C**) Antineoplastic (anticancer) agents per target. (**D**) Stepwise narrowing of the catalog from all unique agents (excluding AR; n = 734) to FDA-approved antineoplastic drugs (n = 219); blood–brain-barrier penetration and CNS pharmacokinetics remain necessary filters for translational prioritization.

Pharmacogenomic mapping of the 176 prioritized candidates using DGIdb v5 (26 curated sources; Supplementary Table S8 and Figure 7A) identified 70 druggable targets. Because AR alone accounts for 899 drug interactions (364 FDA-approved unique drugs) and inflates catalog-level totals, we report counts both with and without AR: excluding AR yields 734 unique agents (230 FDA-approved) across 69 druggable targets, which we treat as the primary GBM-relevant pharmacogenomic space. We emphasize that the great majority of these agents were approved for indications unrelated to GBM and are not proposed as candidate therapies; this mapping is a catalog-level, hypothesis-generating resource, and translational prioritization would require filtering for CNS activity, blood–brain-barrier penetration, and target relevance in glioma. Including AR, the full mapping comprises 1583 unique drugs (554 FDA-approved) across 1722 drug–gene interactions, partitioned into mechanistic categories: inhibitors (204), agonists (94), modulators (52), blockers (7), binders (5), antibodies (3), antibody–inhibitor (1), cleavage (2), activators (1), negative modulators (4), and unknown/unclassified mechanism (1348 ‘unknown’ + 1 ‘other/unknown’ = 1349) (Figure 7B); anticancer (antineoplastic) agents per target and the stepwise narrowing from the full catalog to FDA-approved antineoplastic agents are shown in Figure 7C and Figure 7D, respectively. Drug-degree rank distribution and the FDA-approval × interaction-type composition are summarized in Supplementary Figure S6E,F.

## 4. Discussion

Prior studies have demonstrated that DAC can potentiate TMZ cytotoxicity by demethylating the MLH1 promoter and restoring mismatch repair (MMR) competence and that DAC-mediated global demethylation re-expresses cancer testis antigens and neoantigens, thereby sensitizing GBM cells to T-cell-mediated killing. Our six-layer integrated analysis identifies a mechanistic framework and a prognostic signature that reframe the rationale for DAC in TMZ-resistant GBM. A central negative finding is that DAC does not broadly reverse the TMZ resistance transcriptome (Spearman ρ = 0.073; Reversal Index Wilcoxon *p* = 0.990; only 8 of 11,707 genes [0.07%] strictly reversed).

The dominant IFN-α and IFN-γ enrichments in resistant cells are biologically relevant: although interferon signaling is canonically anti-tumoral, sustained tumor-cell-intrinsic ISG expression can paradoxically promote survival, drive immune exclusion, and confer chemotherapy resistance [37]. The composite identity we observe—concurrent ISG, TNFα/NF-κB, and IL-6/JAK/STAT3 enrichment, alongside reciprocal Myc V1/V2 suppression—resembles the ISG15-high tumor-associated macrophage subtype recently described in GBM single-cell studies and is consistent with a transition from a Myc-driven cycling state to a slower-cycling, stress-adapted, resistant phenotype. Co-upregulation of CHI3L1 and SERPINA1 reinforces a mesenchymal-inflammatory identity, both being established markers of the mesenchymal GBM subtype that correlate with adverse prognosis.

CTA reactivation by demethylating agents is a recognized mechanism for enhancing immunogenic visibility, demonstrated directly in GBM by Ma et al.; our analysis identifies the specific CTA repertoire reactivated across independent GBM systems [12], and, at single-cell resolution in these same DAC-treatment datasets, by Lai et al. [16]. Unlike these prior reports, our analysis formally tests whether this DAC-induced program constitutes reversal of an independently defined TMZ-resistance transcriptome, a question neither study addressed [16,17]. The strictly reversed gene RARB (TMZ log_2_FC = +1.31; DAC log_2_FC = −0.66) is mechanistically counterintuitive given its tumor-suppressor role and warrants functional validation before retinoid combination strategies are pursued [38]. Among direct silencing targets, the paradoxical DAC-induced hypermethylation at S100A4 points to indirect chromatin regulatory effects that may contribute to the MES-like attenuation observed in single-cell data [39].

The mesenchymal dominance observed in our resistant models is consistent with NF-κB activation and AP-1-driven mesenchymal transition, as described in the recurrent GBM [40]. DAC-induced reduction in per-cell MES-like transcriptional intensity in cells already selected for TMZ resistance suggests that DAC can partially oppose mesenchymal transition even after it has become established, a plausible mechanism because the MES-like state is regulated by NF1 loss and NF-κB activation, both of which are sensitive to epigenetic modulation. The IFN-response state’s largest UMAP displacement (d = 3.84) and concordance of state shifts across both GS025 and GS243 models are consistent with, but do not by themselves establish generalizability across heterogeneous GBM contexts. The co-enrichment of IFN/innate immunity and mesenchymal/inflammatory pathways defines a convergent IFN–EMT axis as the central biological theme unifying the prioritized candidates.

The DAC + PARP-inhibitor combination has computational rationale only in this study, and wet-lab validation of PARP trapping and synthetic lethality is required. Mechanistically, DNMT inhibition-mediated demethylation can generate replication stress and DNA strand breaks that, in other tumor systems, increase dependence on PARP-mediated repair [41]. This mechanism is distinct from classical BRCA-mutant synthetic lethality and is therefore applicable to the BRCA-proficient GBM context [42]. The anti-ICAM1 strategies (enlimomab, natalizumab) are worth considering given ICAM1’s multi-layer evidence support across L1 (TMZ resistance), L2 (DAC response), L5 (state transition), and L6 (transcription factor) layers and its individual prognostic significance in TCGA-GBM univariate Cox analysis (HR = 1.28, *p* = 0.011; Supplementary Table S7) [43,44]. Among these, DAC combined with immune checkpoint inhibitors represents perhaps the most tractable combinatorial hypothesis for future testing, supported by CTA reactivation, interferon pathway induction, and prior evidence for DAC-mediated immunosensitization; BBB penetration constraints are discussed in the Limitations section. This study makes three principal contributions: a quantitative demonstration that DAC does not globally reverse the TMZ resistance transcriptome (ρ = 0.073; 0.07% strict reversal), reframing its mechanistic rationale toward complementary epigenetic modes of action; identification of INPP5D/SHIP1 as the top direct epigenetic-reactivation target, defining a putative PI3K/AKT axis independent of DAC’s immune-activating effects; and an 11-gene TCGA-validated prognostic model derived from the six-layer DAC-responsive signature that bridges experimental pharmacology and clinical outcome prediction. Importantly, all combinations proposed here are computational hypotheses for future preclinical testing, not clinical recommendations. Their feasibility in GBM will further depend on factors this analysis does not address: blood–brain-barrier penetration and CNS pharmacokinetics (decitabine itself has limited CNS bioavailability and a short plasma half-life), on-target and myelosuppressive toxicity, achievable intratumoral drug exposure, and the tolerability of multi-agent regimens in a neuro-oncology population.

### Limitations and Future Directions

This study has several limitations. First, all analyses are computational, based on publicly available data, and require functional validation. The TMZ-resistance dataset (GSE151680) was originally generated by Liu et al. [17] to construct and externally validate a ferroptosis-related prognostic signature in glioma; the present study re-purposes these raw expression data to define an independent, IFN/mesenchymal-driven TMZ-resistance transcriptional state and formally test its reversal by DAC, an application and analytical framework distinct from the original ferroptosis-focused report. The TMZ resistance dataset (GSE151680) includes U87MG, a cell line whose genomic authenticity as a GBM model has been questioned because it lacks canonical GBM-defining features such as EGFR amplification and chromosome 10 loss [45], although the high intra-group transcriptional correlation (r ≥ 0.92) between U87MG and U251MG suggests concordant resistance programs. Cell-line-stratified sensitivity analyses showed that 95.5% of the 627-gene TMZ-resistance signature is recovered in a U251MG-only re-derivation, and 87.2% is recovered in a U87MG-only re-derivation (Figure 1F; Supplementary Table S1), confirming that the resistance signature is not driven by either cell line alone. In addition, U87MG and U251MG are long-established serum-adapted lines that do not fully reflect the current histopathological and molecular definition of GBM; patient-derived, IDH-wildtype models with confirmed genomic authenticity will be needed to consolidate these findings. Future studies should nevertheless validate this signature in patient-derived GBM stem cell models with confirmed genomic authenticity. Second, the reversal analysis was performed at the bulk transcriptome level, and it remains possible that reversal events occur at a cell-state-specific or subpopulation level not captured in bulk data. The single-cell dataset (GSE261188), like the companion DAC bulk RNA-seq (GSE261187) and methylation (GSE261190) datasets, was originally generated by Lai et al. [16] to characterize DAC-induced immunogenic reprogramming rather than TMZ-resistance reversal, and was derived from only two GSC models (GS025 and GS243), limiting its generalizability across the full spectrum of GBM heterogeneity; future studies using patient-derived organoid systems or in vivo models would provide more clinically relevant context.

Third, the 11-gene prognostic model was developed and internally validated in the TCGA-GBM cohort (n = 166 patients with complete OS data). External validation in an independent cohort such as CGGA, REMBRANDT, or the GLASS Consortium is essential before any clinical utility can be claimed, and the modest sample size and internal-only validation mean that overfitting cannot be excluded despite LASSO regularization [46,47]. In this revision we quantified this optimism directly: repeating the full two-stage selection inside a bootstrap raised the optimism estimate to 0.062 (selection-inclusive optimism-corrected C-index ~0.63), confirming that the apparent performance is optimistic. We then performed external validation in the independent CPTAC-GBM cohort (n = 96): the frozen 11-gene model achieved only a C-index of 0.55 (95% CI 0.47–0.63; tertile log-rank *p* = 0.33), confirming that the signature does not generalize to an independent cohort and is best interpreted as an exploratory, hypothesis-generating tool rather than a prognostic biomarker. External validation in CGGA, REMBRANDT, or GLASS is the single most important next step for translational readiness; the GSE80137 four-of-five signature replication, given that GSE80137 also contributed to the 665-gene DAC consensus, reflects signature robustness rather than independent external validation. Fourth, methylation data were derived from the EPIC 850K array rather than whole-genome bisulfite sequencing (WGBS) and may not fully capture regulatory elements outside canonical promoter CpG islands. Integration of datasets from different platforms and laboratories may introduce residual batch effects despite normalization. Fifth, the pharmacogenomic analysis does not incorporate blood–brain barrier penetration or pharmacokinetic data, critical practical considerations for CNS drug development that constrain the translational applicability of identified agents [48]. Priority next steps are functional validation of INPP5D reactivation in MGMT-stratified GBM models, prospective validation of the 11-gene prognostic model in CGGA/REMBRANDT/GLASS cohorts, preclinical testing of DAC + PARP and DAC + ICI combinations, and spatial transcriptomic profiling of DAC-induced state transitions in patient-derived tissue.

## 5. Conclusions

This integrative multi-omics analysis provides a systematic molecular characterization of the interplay between DAC and TMZ resistance in GBM. DAC does not broadly reverse the TMZ-resistance transcriptome in the datasets and models analyzed here (11,707 genes; rho = 0.073) but acts through three mechanistically distinct programs: epigenetic reactivation of tumor-suppressive and immunogenic loci including a putative INPP5D/SHIP1–PI3K/AKT axis; per-cell attenuation of mesenchymal and stem-like transcriptional intensity coupled to population-level depletion of cycling and IFN-response compartments; and induction of a cancer-testis-antigen/interferon program consistent with viral mimicry biology. Six-layer integration prioritized 176 candidates, unified by a convergent interferon–EMT axis, reframing DAC as a transcriptional reprogramming agent rather than a direct reversal agent, and providing a molecular rationale for combination strategies targeting this axis. These conclusions are computational and hypothesis-generating: the study supports a model of DAC-mediated reprogramming but does not demonstrate therapeutic efficacy, which will require functional and clinical validation.

## Figures and Tables

**Table 1:** Public datasets analyzed in this study.

Dataset ID	Platform	Condition	n	Source
GSE151680	Illumina RNA-seq	TMZ-resistant vs. sensitive	12	GEO
GSE261187	Illumina RNA-seq	DAC-treated vs. control	6	GEO
GSE80137	Illumina RNA-seq	DAC-treated vs. control	8	GEO
GSE261190	EPIC 850K methylation	DAC-treated vs. control	12	GEO
GSE261188	10× scRNA-seq	DAC-treated vs. control	29,769 cells	GEO
TCGA-GBM	RNA-seq + clinical	OS, vital status	166	UCSC Xena
DGIdb v5	Drug-gene database	Curated interactions	N/A	Online

## Data Availability

The original data presented in the study are openly available in the Gene Expression Omnibus (GEO) at https://www.ncbi.nlm.nih.gov/geo/ (accessed on 10 July 2026) under accessions GSE151680, GSE261187, GSE80137, GSE261190, and GSE261188. TCGA-GBM data are available through the UCSC Xena Browser (https://xenabrowser.net/, accessed on 10 July 2026). All analysis code and processed data tables will be made available on GitHub upon acceptance.

## Supplementary Materials

The following supporting information can be downloaded at: https://www.mdpi.com/article/10.3390/cancers18162616/s1, Figure S1: DE-method robustness and sample correlation. (**A**) 3 × 3 effect-size correlation heatmap across limma-trend/limma-aw/Wilcoxon (n = 15,469 genes). (**B**) limma-trend vs. limma-aw logFC hexbin (Pearson r = 0.991). (**C**) 3-method UpSet of significant DEG intersections. (**D**) Top-15 DEGs parallel coordinates. (**E**) MA plot (limma-aw) and (**F**) Top-N DEG recovery matrix; Figure S2: DAC transcriptional reprogramming and reversal-analysis diagnostics. (**A**) Per-dataset DAC logFC hexbin (GSE261187 × GSE80137; n = 16,482; ρ = 0.384). (**B**) Direction-concordance contingency heatmap. (**C**) Per-sample Reversal Index split violin. (**D**) GSEA enrichment of TMZ-resistance signatures on DAC-ranked gene lists (TMZ_Resistance_UP p.adj = 4.0 × 10^−11^; TMZ_Resistance_ALL p.adj = 2.7 × 10^−9^; TMZ_Resistance_DOWN p.adj = 2.0 × 10^−1^). (**E**) Sensitivity analysis showing DAC consensus gene yield across four threshold tiers (Ultra-strict, Strict, Moderate, Relaxed; n = 116, 473, 523, and 739 up-DEGs, and 0, 75, 253, and 418 down-DEGs, respectively) and (**F**) Cross-dataset DAC consensus funnel: 16,482 common-tested genes → 1957 significant in either (11.9%) → 23 significant in both (1.2%) → 228 both + concordant → 665 final consensus DAC set, representing a 2.92× expansion over the 228-gene strict-overlap pool (equivalently +191.7%; the “291.7%” arrow label in the figure denotes 665 as a percentage of 228) (Pearson r = 0.4254, Spearman ρ = 0.3840); Figure S3: DNA methylation quality control and MGMT probe analysis. (**A**) Per-array EPIC QC scatter (20 arrays). (**B**) DMP density volcano (803,785 CpGs; 201,434 hypomethylated + 174,949 hypermethylated at FDR < 0.05; the strict cut |Δβ| > 0.10 retains 175,180 DMPs). (**C**) Δβ ridge by genomic region. (**D**) MGMT 191-probe Δβ strip by region. (**E**) Region-stratified DMP volcano and (**F**) Integration-class donut; Figure S4: Microglial marker deconvolution. (**A**) INPP5D gene model with per-probe Δβ across 66 EPIC probes (red = significant strict; teal = significant relaxed). (**B**) Paired DMSO → DAC β per probe (mean β_DMSO = 0.733; mean β_DAC = 0.668; mean Δβ = −0.066; 42/66 hypomethylated). (**C**) Methylation × expression plane (Δβ vs. RNA log_2_FC) for 14,443 genes with INPP5D highlighted as the rank #1 epigenetic activation target and (**D**) INPP5D probe-level volcano (Δβ vs. −log_10_ p) coloured by CpG-island context; Figure S5: Single-cell QC and state-composition diagnostics. (**A**) Per-cell UMI × gene QC hexbin (n = 29,769 pre-filter; 21,676 post-filter). (**B**) Per-sample mitochondrial percentage ridge plot. (**C**) Mesenchymal × Cycling module-score scatter showing all 21,676 cells coloured by Seurat cluster, with the 8 state centroids overlaid as labelled dots; labels for the seven non-Cycling states cluster near the origin reflecting their lower mean Mesenchymal/Cycling module scores relative to the Mesenchymal- and Cycling-dominated states; per-state mean coordinates. (**D**) Top-3 marker genes per Seurat cluster (0–11) shown as dot plot; color = average log_2_FC, size = expression frequency and (**E**) Module-score violins per cell state, split DMSO vs. DAC; significant differences marked (***); Figure S6: Machine-learning and TCGA-GBM survival diagnostics. (**A**) ML3 10-fold cross-validation R^2^ per fold (mean = 0.019, SD = 0.018). (**B**) Per-gene expression density (z-score) across 166 TCGA-GBM samples for the 11 prognostic genes (AEBP1, ANK1, LOXL1, G0S2, CD81, NNAT, DKK3, PTTG1, HOXB3, TIMP4, AR). (**C**) Pairwise Pearson correlation matrix of the 11 prognostic genes. (**D**) 11-gene risk-score distribution per tertile (Low/Med/High) with event vs. censored markers. (**E**) DGIdb drug-degree rank distribution (all drugs vs. FDA-approved; AR labeled as the highest-degree target) and (**F**) DGIdb 2-level sunburst (inner = FDA-approved vs not; outer = interaction-type composition).; Figure S7: Robustness of the six-layer integration score (label-permutation null, leave-one-layer-out sensitivity, and full-pipeline bootstrap) and internal validation of the 11-gene survival model (bootstrap C-index, univariate Cox, Kaplan–Meier, and two-stage-selection optimism). (**A**) Genes per evidence layer (L1–L6). (**B**) Genes by number of supporting layers (≥4-layer core = 12). (**C**) DAC vs. TMZ effect sizes (ρ = 0.073). (**D**) Observed vs. permutation-null convergence per threshold (≥4 layers ~18×, *p* < 1 × 10^−4^; ≥3 layers *p* = 0.19). (**E**) Leave-one-layer-out Jaccard overlap. (**F**) Bootstrap gene-selection frequency (★ = signature). (**G**) 11-gene expression correlation (TCGA-GBM). (**H**) Model coefficients. (**I**) Univariate Cox HRs. (**J**) Kaplan–Meier by risk tertile (log-rank *p* = 6 × 10^−10^). (**K**) Bootstrap C-index (0.706; 95% CI 0.66–0.75) and (**L**) Optimism-corrected C-index (final 0.676; with selection 0.63).; Figure S8: External validation of the 11-gene model in the independent CPTAC-GBM cohort (near-chance discrimination). (**A**) Kaplan–Meier by risk tertile (log-rank *p* = 0.33, n.s.). (**B**) Bootstrap external C-index (0.55; 95% CI 0.47–0.63, includes 0.50). (**C**) C-index in TCGA (apparent 0.706; optimism-corrected 0.630) vs. CPTAC (0.549). (**D**) Per-gene hazard ratios, TCGA vs. CPTAC (effect sizes collapse toward HR ≈ 1). (**E**) CPTAC univariate Cox HRs (per SD); none significant and (**F**) Risk score by vital status (overlapping). External discrimination is near chance, indicating the signature does not generalize and is exploratory; Table S1: TMZ-resistance DEGs and U251MG-only sensitivity re-derivation; Table S2: 665 DAC consensus-responsive genes; Table S3: 146 direct epigenetic targets (sheet S3_DirectEpiTargets_146) and 191-probe MGMT EPIC methylation (sheet S3_MGMT_EPIC_191probes); Table S4: 43-marker cell-type deconvolution panel; Table S5: 176 prioritized Tier-1/Tier-2 candidates; Table S6: GENIE3 network hub statistics and JUN in/out-degree reconciliation; Table S7: ML3 10-fold per-fold R^2^, TCGA-GBM univariate Cox, 11-gene per-patient risk scores, and decile-level calibration; Table S8: DGIdb v5 drug–target interactions (1722 rows, 1583 unique drugs, 554 FDA-approved).

## Abbreviations

AKT	AKT serine/threonine kinase (protein kinase B)
AUC	Area under the curve
BBB	Blood–brain barrier
BH	Benjamini–Hochberg
BMIQ	Beta-mixture quantile normalization
C-index	Concordance index
CGGA	Chinese Glioma Genome Atlas
CI	Confidence interval
CNS	Central nervous system
CpG	Cytosine–phosphate–guanine dinucleotide
CTA	Cancer-testis antigen
CV	Cross-validation
DAC	Decitabine (5-aza-2′-deoxycytidine)
DEG	Differentially expressed gene
DGIdb	Drug–Gene Interaction Database
DMP	Differentially methylated position
DMSO	Dimethyl sulfoxide
DNMT	DNA methyltransferase
DoRothEA	Discriminant Regulon Expression Analysis
EMT	Epithelial–mesenchymal transition
EPIC	Infinium MethylationEPIC BeadChip (850K methylation array)
ERV	Endogenous retrovirus
FDA	U.S. Food and Drug Administration
FDR	False discovery rate
GBM	Glioblastoma
GENIE3	Gene Network Inference with Ensemble of trees
GEO	Gene Expression Omnibus
GLASS	Glioma Longitudinal Analysis Consortium
GSC	Glioma stem cell
GSEA	Gene set enrichment analysis
GSVA	Gene set variation analysis
HR	Hazard ratio
HTSeq	High-throughput sequencing (read-counting framework)
ICAM1	Intercellular adhesion molecule 1
IFN	Interferon
IQR	Interquartile range
ISG	Interferon-stimulated gene
JAK	Janus kinase
KDE	Kernel density estimation
KEGG	Kyoto Encyclopedia of Genes and Genomes
LASSO	Least absolute shrinkage and selection operator
MGMT	O6-methylguanine–DNA methyltransferase
MMR	DNA mismatch repair
MSigDB	Molecular Signatures Database
mTORC1	Mechanistic target of rapamycin complex 1
NES	Normalized enrichment score
NF-κB	Nuclear factor kappa B
OS	Overall survival
PAGA	Partition-based graph abstraction
PARP	Poly(ADP-ribose) polymerase
PCA	Principal component analysis
PI3K	Phosphoinositide 3-kinase
QC	Quality control
REMBRANDT	Repository for Molecular Brain Neoplasia Data
RI	Reversal Index
RNA-seq	RNA sequencing
ROC	Receiver operating characteristic
SD	Standard deviation
SEM	Standard error of the mean
SNR	Signal-to-noise ratio
STAR	Spliced Transcripts Alignment to a Reference
STAT	Signal transducer and activator of transcription
TCGA	The Cancer Genome Atlas
TCGA-GBM	The Cancer Genome Atlas Glioblastoma cohort
TF	Transcription factor
TMM	Trimmed mean of M-values
TMZ	Temozolomide
TSS	Transcription start site
UCSC	University of California, Santa Cruz (Xena Browser)
UMAP	Uniform manifold approximation and projection
UTR	Untranslated region
VIPER	Virtual Inference of Protein activity by Enriched Regulon analysis
WGBS	Whole-genome bisulfite sequencing
