# Supplementary material for: Decitabine Reprograms Temozolomide-Resistant Glioblastoma Through Epigenetic Reactivation and Mesenchymal Attenuation: A Multi-Omics Study
Source: Cancers (Basel). 2026 Aug 14;18(16):2616. doi: 10.3390/cancers18162616 (PMC13511076; doi:10.3390/cancers18162616)
Supplement: Supplementary file 1 [file cancers-18-02616-s001.zip › Supplementary Figures.pdf]

Decitabine Reprograms Temozolomide-Resistant Glioblastoma Through Epigenetic Reactivation and Mesenchymal Attenuation: A Multi-Omics Study

Itika Arora [1], Shamsa Hilal Saleh [1], Arshiya Akbar [1], Fareeha Arshad [1], Volodymyr Mavrych [1], Olena Bolgova [1], Faisal Abdulhameed Farrash [2], Ahmed Abu-Zaid [1], Andleeb Khan [3,4], Sheikh Muskan [3,4], Mohammed Imran Khan [5,*] and Ahmed Yaqinuddin [1,*]

[1] College of Medicine, Alfaisal University, Riyadh 11533, Saudi Arabia
[2] King Faisal Specialist Hospital and Research Centre (KFSHRC), Riyadh 12713, Saudi Arabia
[3] Department of Biosciences, Faculty of Science, Integral University, Lucknow 226026, India
[4] Stem Cell Biology Lab, Integral Center of Excellence for Interdisciplinary Research-4 (ICEIR-12), Integral University, Lucknow 226026, India
[5] King Faisal Specialist Hospital and Research Centre (KFSHRC), Jeddah 23433, Saudi Arabia
* Correspondence: mikhan@kfshrc.edu.sa (M.I.K.); ayaqinuddin@alfaisal.edu (A.Y.)

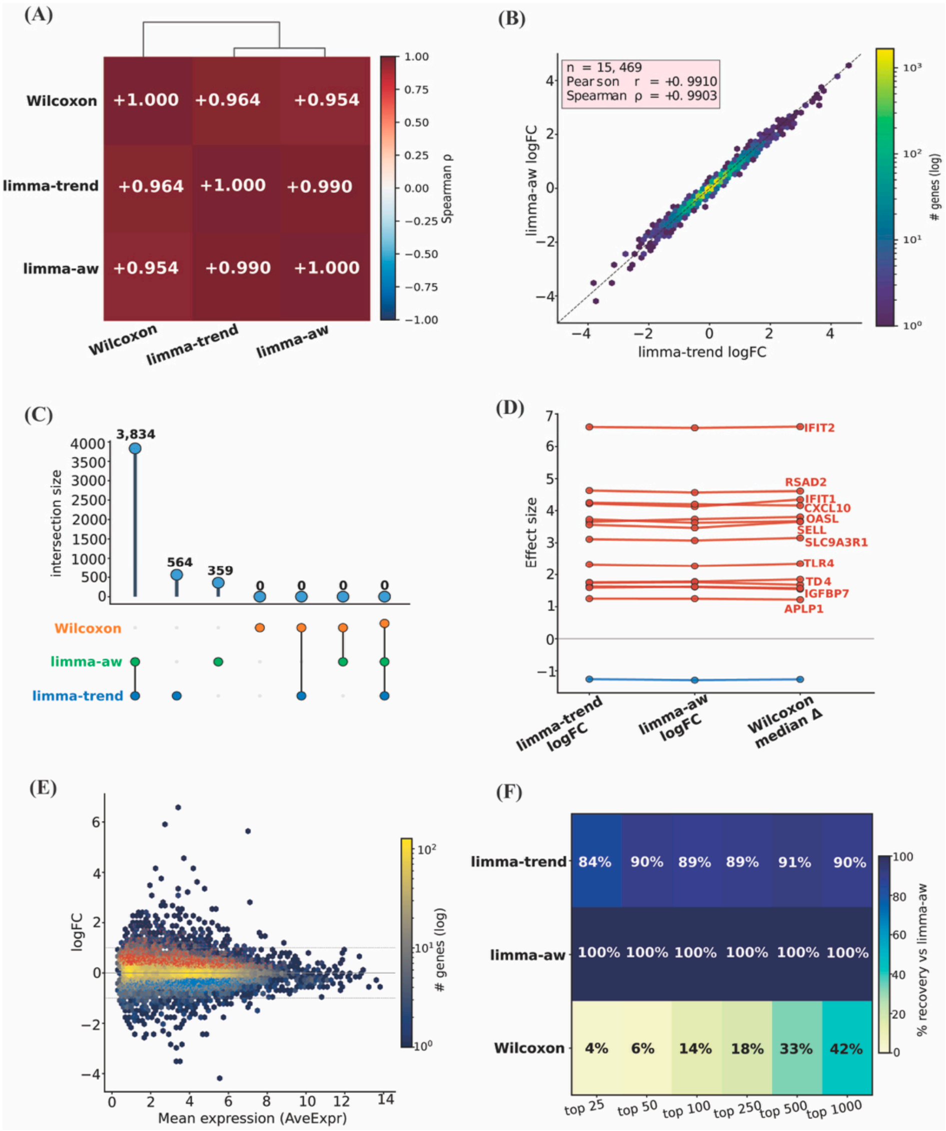

Supplementary Figure S1. DE-method robustness and sample correlation. (A) 3 × 3 effect-size correlation heatmap across limma-trend / limma-aw / Wilcoxon (n = 15,469 genes). **(B)** limma-trend vs limma-aw logFC hexbin (Pearson r = 0.991). (C) 3-method UpSet of significant DEG intersections. **(D)** Top-15 DEGs parallel coordinates. **(E)** MA plot (limma-aw) and **(F)** Top-N DEG recovery matrix.

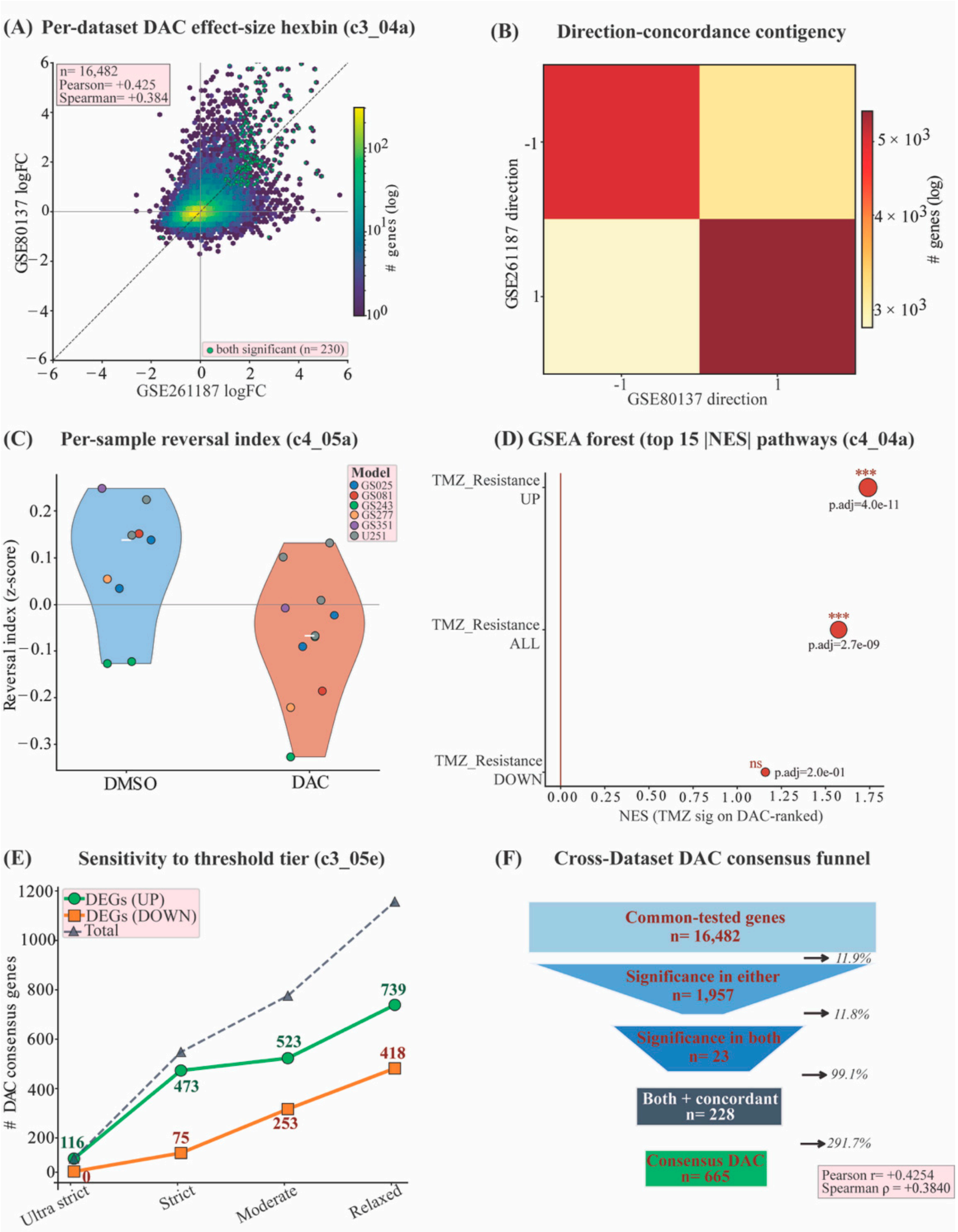

Supplementary Figure S2. DAC transcriptional reprogramming and reversal-analysis diagnostics. (A) Per-dataset DAC logFC hexbin (GSE261187 × GSE80137; n = 16,482; ρ = 0.384). **(B)** Direction-concordance contingency heatmap. **(C)** Per-sample Reversal Index split violin. **(D)** GSEA enrichment of TMZ-resistance signatures on DAC-ranked gene lists (TMZ_Resistance_UP p.adj = 4.0×10^{-11}; TMZ_Resistance_ALL p.adj = 2.7×10^{-9}; TMZ_Resistance_DOWN p.adj = 2.0×10^{-1}). **(E)** Sensitivity analysis showing DAC consensus gene yield across four threshold tiers (Ultra-strict, Strict, Moderate, Relaxed; n = 116, 473, 523, and 739 up-DEGs, and 0, 75, 253, and 418 down-DEGs, respectively) and **(F)** Cross-dataset DAC consensus funnel: 16,482 common-tested genes → 1,957 significant in either (11.9%) → 23 significant in both (1.2%) → 228 both + concordant → 665 final consensus DAC set, representing a 2.92× expansion over the 228-gene strict-overlap pool (equivalently +191.7%; the "291.7%" arrow label in the figure denotes 665 as a percentage of 228) (Pearson r = 0.4254, Spearman ρ = 0.3840).

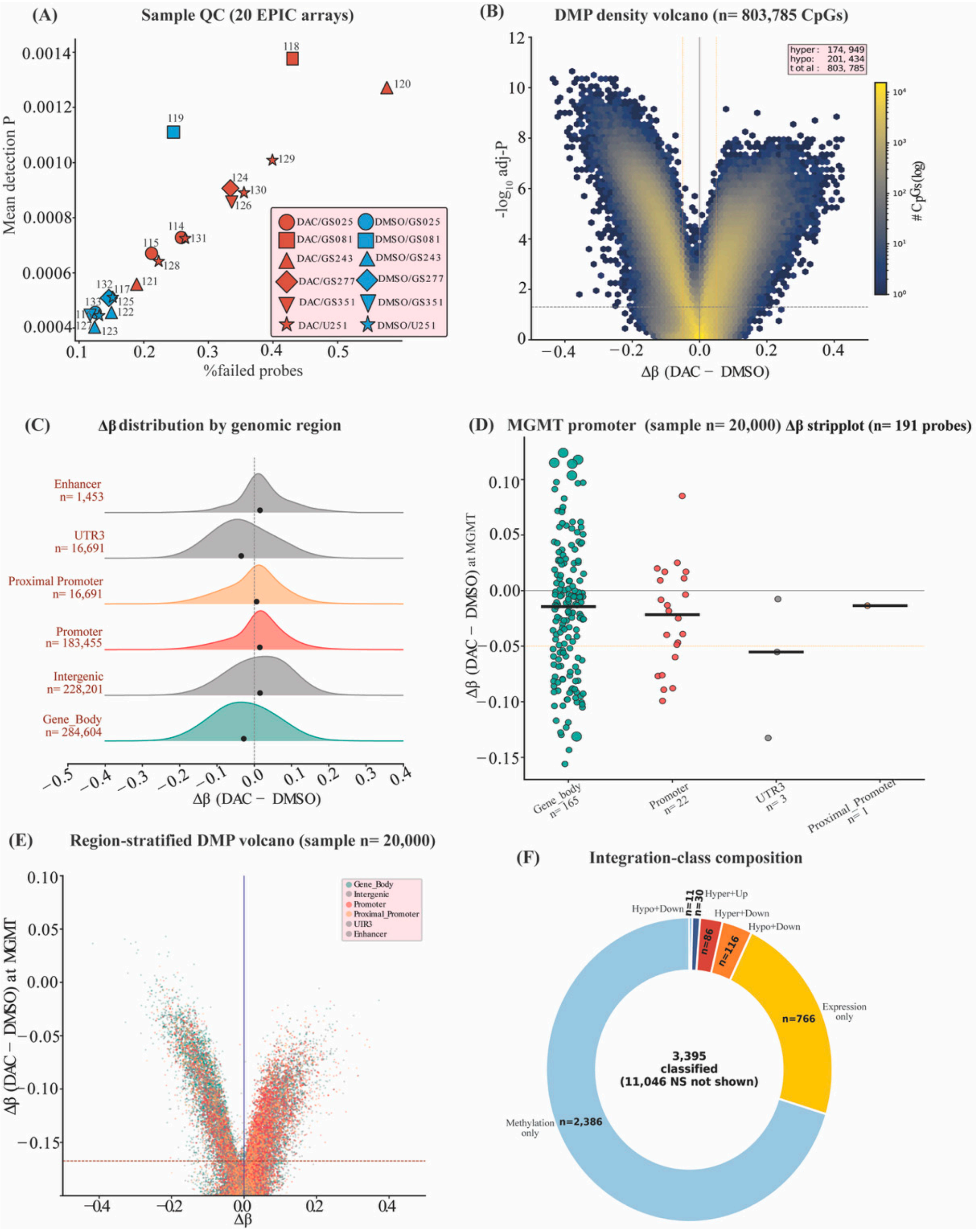

Supplementary Figure S3. DNA methylation quality control and MGMT probe analysis. (A) Per-array EPIC QC scatter (20 arrays). **(B)** DMP density volcano (803,785 CpGs; 201,434 hypomethylated + 174,949 hypermethylated at FDR < 0.05; the strict cut |Δβ| > 0.10 retains 175,180 DMPs). **(C)** Δβ ridge by genomic region. (D) MGMT 191-probe Δβ strip by region. **(E)** Region-stratified DMP volcano and **(F)** Integration-class donut.

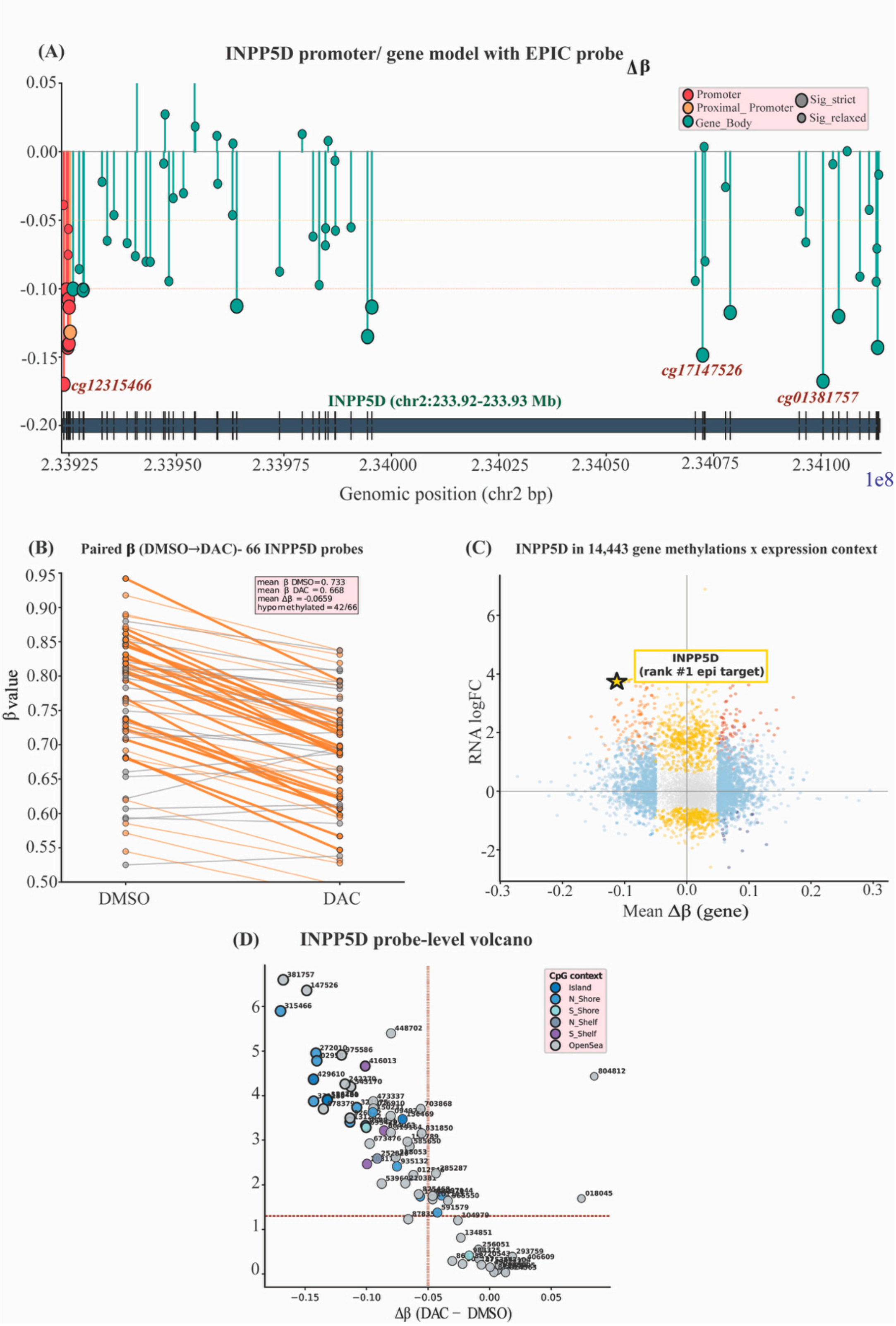

Supplementary Figure S4. Microglial marker deconvolution. (A) INPP5D gene model with per-probe Δβ across 66 EPIC probes (red = significant strict; teal = significant relaxed). **(B)** Paired DMSO → DAC β per probe (mean β_DMSO = 0.733; mean β_DAC = 0.668; mean Δβ = −0.066; 42/66 hypomethylated). **(C)** Methylation × expression plane (Δβ vs RNA $\log_2$FC) for 14,443 genes with INPP5D highlighted as the rank #1

epigenetic activation target and **(D)** INPP5D probe-level volcano ($\Delta\beta$ vs $-\log_{10}$ p) coloured by CpG-island context.

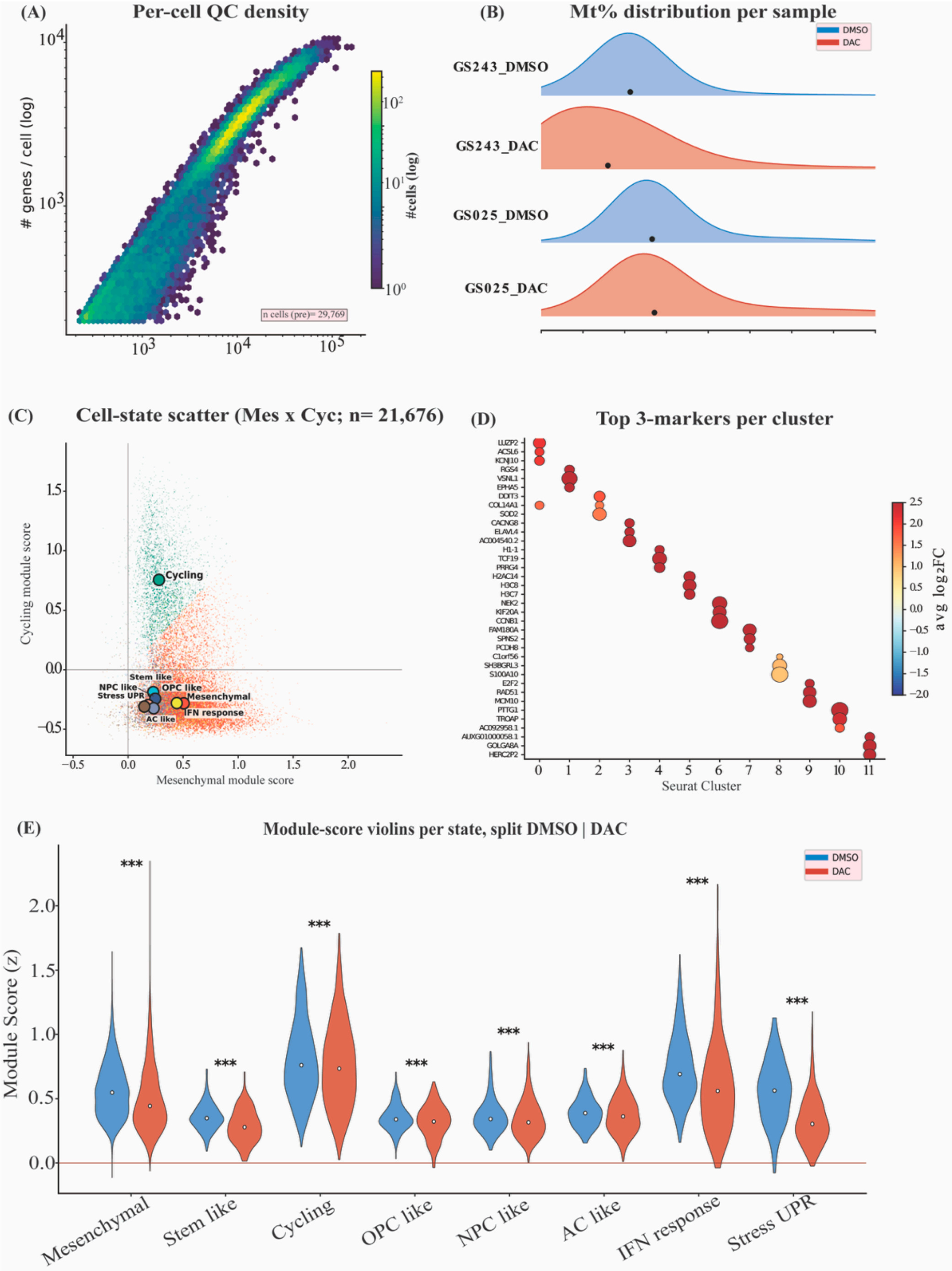

Supplementary Figure S5. Single-cell QC and state-composition diagnostics. **(A)** Per-cell UMI × gene QC hexbin (n = 29,769 pre-filter; 21,676 post-filter). **(B)** Per-sample mitochondrial percentage ridge plot. **(C)** Mesenchymal × Cycling module-score scatter showing all 21,676 cells coloured by Seurat cluster, with the 8 state centroids overlaid as labelled dots; labels for the seven non-Cycling states cluster near the origin reflecting their lower mean Mesenchymal/Cycling module scores relative to the Mesenchymal- and Cycling-dominated states; per-state mean coordinates. **(D)** Top-3 marker genes per Seurat cluster (0–11) shown as dot plot;

color = average log_2FC, size = expression frequency and **(E)** Module-score violins per cell state, split DMSO vs DAC; significant differences marked (***).

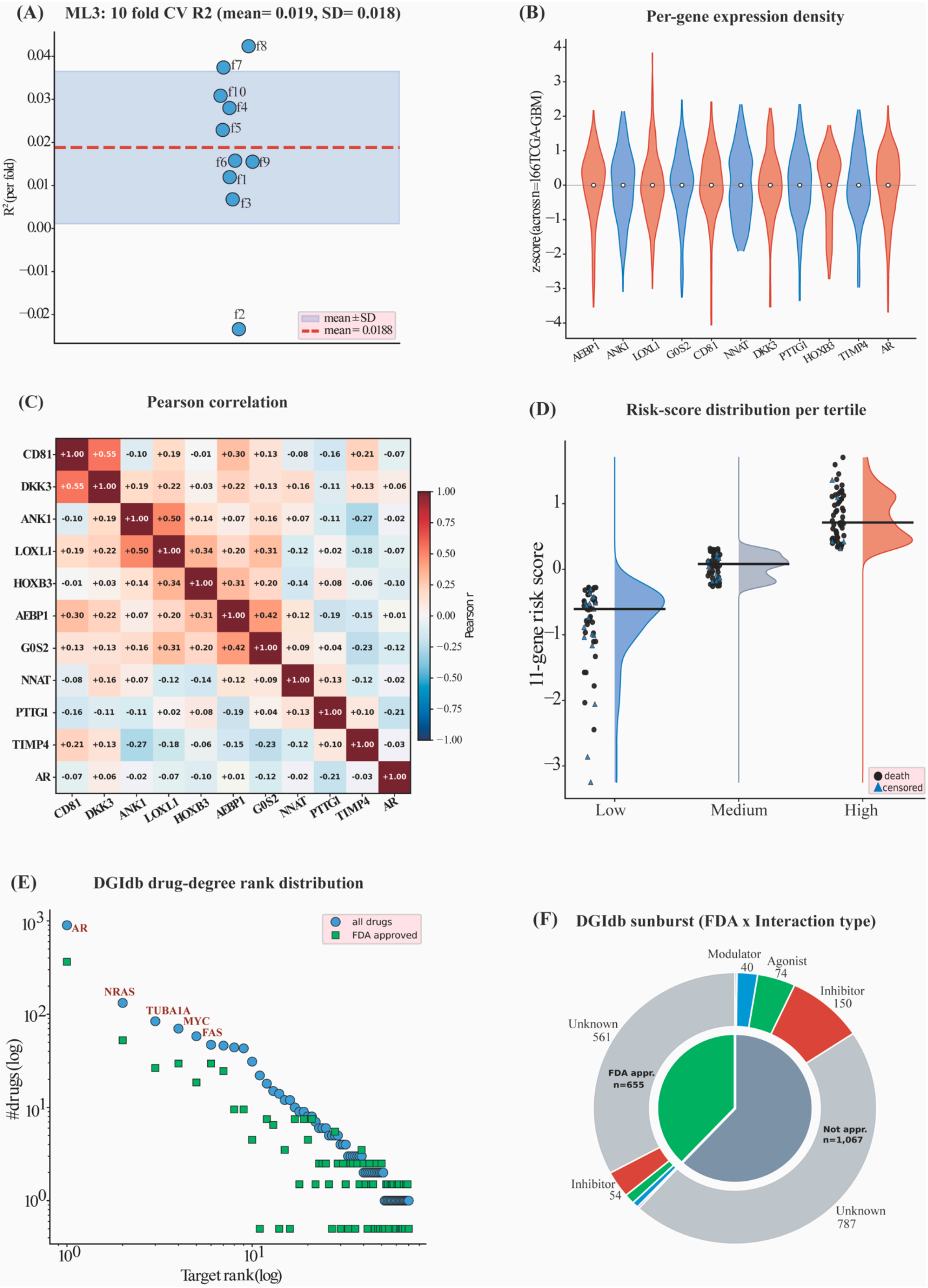

Supplementary Figure S6. Machine-learning and TCGA-GBM survival diagnostics. (A) ML3 10-fold cross-validation R^2 per fold (mean = 0.019, SD = 0.018). **(B)** Per-gene expression density (z-score) across 166 TCGA-GBM samples for the 11 prognostic genes (AEBP1, ANK1, LOXL1, G0S2, CD81, NNAT, DKK3, PTTG1, HOXB3, TIMP4, AR). **(C)** Pairwise Pearson correlation matrix of the 11 prognostic genes. **(D)** 11-gene risk-score distribution per tertile (Low/Med/High) with event vs censored markers. **(E)** DGIdb drug-degree rank distribution (all drugs vs FDA-approved; AR labeled as the highest-degree target) and **(F)** DGIdb 2-level sunburst (inner = FDA-approved vs not; outer = interaction-type composition).

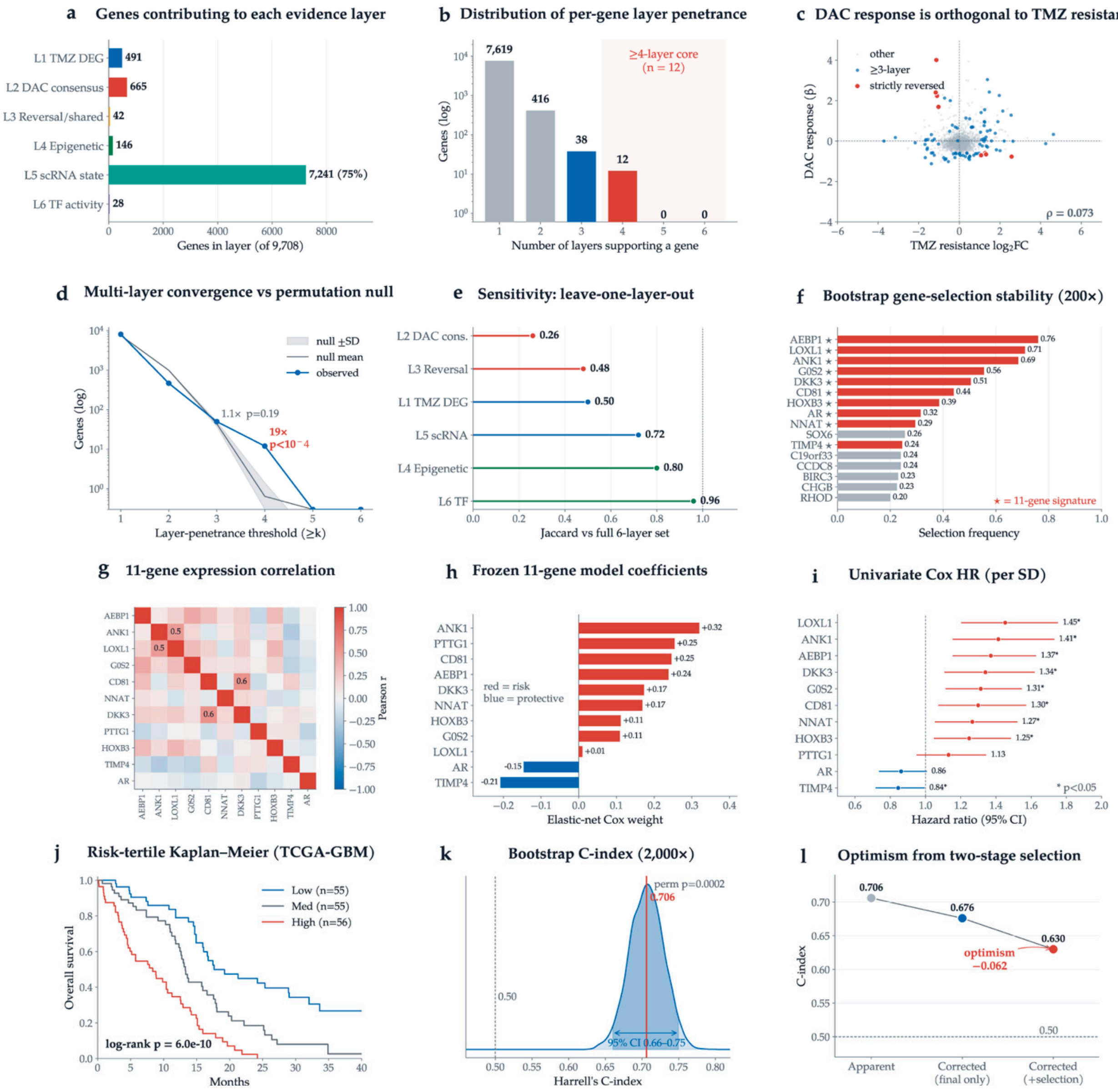

Supplementary Figure S7. Robustness of the six-layer integration score (label-permutation null, leave-one-layer-out sensitivity, and full-pipeline bootstrap) and internal validation of the 11-gene survival model (bootstrap C-index, univariate Cox, Kaplan–Meier, and two-stage-selection optimism). (A) Genes per evidence layer (L1–L6). (B) Genes by number of supporting layers (≥4-layer core = 12). (C) DAC vs TMZ effect sizes (ρ = 0.073). (D) Observed vs permutation-null convergence per threshold (≥4 layers ~18×, $p < 1 \times 10^{-4}$; ≥3 layers p = 0.19). (E) Leave-one-layer-out Jaccard overlap. (F) Bootstrap gene-selection frequency (★ =

signature). (G) 11-gene expression correlation (TCGA-GBM). (H) Model coefficients. (I) Univariate Cox HRs. (J) Kaplan–Meier by risk tertile (log-rank $p = 6 \times 10^{-10}$). (K) Bootstrap C-index (0.706; 95% CI 0.66–0.75) and (L) Optimism-corrected C-index (final 0.676; with selection 0.63).

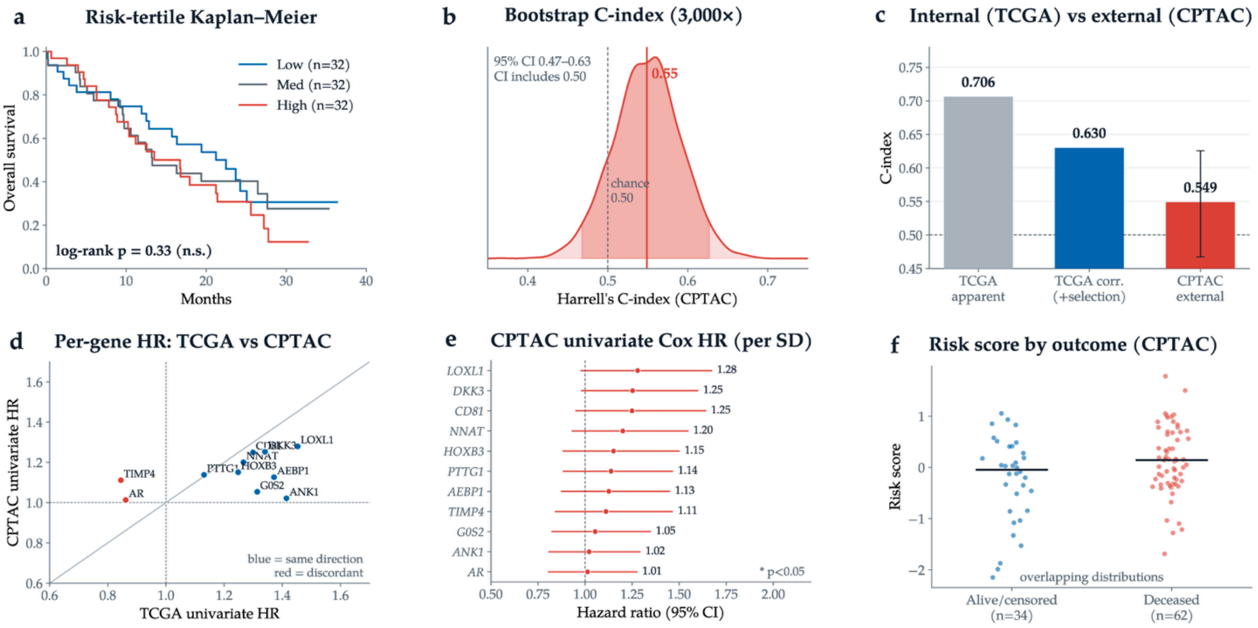

Supplementary Figure S8. External validation of the 11-gene model in the independent CPTAC-GBM cohort (near-chance discrimination). (A) Kaplan–Meier by risk tertile (log-rank p = 0.33, n.s.). (B) Bootstrap external C-index (0.55; 95% CI 0.47–0.63, includes 0.50). (C) C-index in TCGA (apparent 0.706; optimism-corrected 0.630) vs CPTAC (0.549). (D) Per-gene hazard ratios, TCGA vs CPTAC (effect sizes collapse toward HR ≈ 1). (E) CPTAC univariate Cox HRs (per SD); none significant and (F) Risk score by vital status (overlapping). External discrimination is near chance, indicating the signature does not generalize and is exploratory.
